# Computational Predictions Provide Insights into the Biology of TAL Effector Target Sites

**DOI:** 10.1371/journal.pcbi.1002962

**Published:** 2013-03-14

**Authors:** Jan Grau, Annett Wolf, Maik Reschke, Ulla Bonas, Stefan Posch, Jens Boch

**Affiliations:** 1 Institute of Computer Science, Martin Luther University Halle–Wittenberg, Halle (Saale), Germany; 2 Department of Genetics, Institute of Biology, Martin Luther University Halle–Wittenberg, Halle (Saale), Germany; Max Planck Institute for Plant Breeding Research, Germany

## Abstract

Transcription activator-like (TAL) effectors are injected into host plant cells by *Xanthomonas* bacteria to function as transcriptional activators for the benefit of the pathogen. The DNA binding domain of TAL effectors is composed of conserved amino acid repeat structures containing repeat-variable diresidues (RVDs) that determine DNA binding specificity. In this paper, we present TALgetter, a new approach for predicting TAL effector target sites based on a statistical model. In contrast to previous approaches, the parameters of TALgetter are estimated from training data computationally. We demonstrate that TALgetter successfully predicts known TAL effector target sites and often yields a greater number of predictions that are consistent with up-regulation in gene expression microarrays than an existing approach, Target Finder of the TALE-NT suite. We study the binding specificities estimated by TALgetter and approve that different RVDs are differently important for transcriptional activation. In subsequent studies, the predictions of TALgetter indicate a previously unreported positional preference of TAL effector target sites relative to the transcription start site. In addition, several TAL effectors are predicted to bind to the TATA-box, which might constitute one general mode of transcriptional activation by TAL effectors. Scrutinizing the predicted target sites of TALgetter, we propose several novel TAL effector virulence targets in rice and sweet orange. TAL-mediated induction of the candidates is supported by gene expression microarrays. Validity of these targets is also supported by functional analogy to known TAL effector targets, by an over-representation of TAL effector targets with similar function, or by a biological function related to pathogen infection. Hence, these predicted TAL effector virulence targets are promising candidates for studying the virulence function of TAL effectors. TALgetter is implemented as part of the open-source Java library Jstacs, and is freely available as a web-application and a command line program.

## Author Summary

While it had already been discovered that transcription activator-like (TAL) effectors from *Xanthomonas* pathogens act as transcription factors in the host plant, deciphering the modular code of DNA binding specificity of TAL effectors in 2009 fascinated the scientific community. This modular code opens the possibility to identify virulence targets of natural TAL effectors in host plants including valuable crops. Knowing these targets deepens our understanding of the role of TAL effectors in virulence. At the same time, it is an opportunity to create resistant plants by destroying TAL effector target sites, indispensable for the pathogen, in plant genomes. However, computational methods are needed to effectively scan full genomes or promoteromes for putative target sites. Hence, we propose TALgetter, a new approach for predicting TAL effector target sites. Using TALgetter, we predict target sites of *Xanthomonas* TAL effectors in the important crop plants rice and sweet orange. Besides novel putative virulence targets of several TAL effectors, we also gain new insights into the biology of TAL effector targeting. The predictions of TALgetter reveal that target sites are preferentially located in the vicinity of the transcription start and that many TAL effectors bind to the TATA-box in the promoters of target genes.

## Introduction

The DNA-binding domain of transcription activator-like (TAL) effectors is unique in its modular DNA-specificity. Natural TAL effectors are potent virulence proteins from plant-pathogenic *Xanthomonas* bacteria that are injected into eukaryotic host cells where they function as transcription factors [1]. Specific DNA-binding of TAL effectors is mediated by highly conserved tandem repeats composed of usually 34 amino acids. Each repeat recognizes one base pair in a contiguous, non-overlapping fashion. DNA-specificity is determined by two amino acids per repeat at position 12 and 13, termed repeat-variable diresidues (RVDs) [2], [3]. Structures of TAL effector-DNA complexes showed that amino acid 13 interacts with the sense strand DNA base whereas amino acid 12 stabilizes the repeat arrangement [4], [5]. Individual RVDs have specificities for individual DNA bases or combinations thereof [2], [3]. Different RVDs contribute differently to the transcriptional activation by TAL effectors [6]. Typically, natural TAL effector target sites are directly preceded by the nucleotide T, while some target sites also have a C or an A at that position [2], [3], [7]–[10]. The modular repeat architecture allows a rearrangement of TAL effector repeats to easily generate any desired DNA-specificity. Accordingly, TAL effectors were adopted as a preferred biotechnology tool for targeted DNA binding [11]–[13]. Fusion of the TAL effector repeat domain with nuclease, activator, and repressor domains yielded highly specific mutagens, gene switches, and repressors, respectively [11]–[21]. Decoding the DNA specificity of TAL effectors also opens the possibility to identify virulence targets of natural TAL effectors in host plants including valuable crops. The computational prediction of TAL effector target sites in host genomes is a key step to provide candidates for subsequent experimental validation.

The recognition of signals in nucleic acid sequences, such as transcription factor binding sites, splice sites, or translation initiation sites, is one of the major fields of computational biology since the seminal work of Berg and von Hippel [22]. Berg and von Hippel propose a statistical-mechanical model where each base pair contributes independently to the total binding affinity of a DNA-binding protein. The same independence assumption is imposed by Stormo *et al.* [23], who use a scoring matrix learned by the perceptron algorithm for predicting translation initiation sites, and Staden [24], who estimates the entries of a position weight matrix as relative frequencies of nucleotides in a training data set.

Berg and von Hippel already note that the independence assumptions of position weight matrices are most likely not satisfied. First order Markov models or weight array matrix models [25], [26] address this issue and additionally model dependencies between neighboring positions of binding sites. Dependencies between neighboring positions are also taken into account by a special profile hidden Markov model proposed by Salama and Stekel [27] for predicting transcription factor binding sites. Higher order Markov models, which capture dependencies on a larger number of adjacent positions, are employed by Grau *et al.* [28] for the prediction of transcription factor binding sites and by Yakhnenko *et al.* [29] for predicting subcellular localization signals.

Dependencies between non-adjacent binding site positions are represented by Bayesian networks [30], Bayesian trees [31], [32], permuted Markov models [33], and Markov random fields [34]. All models capturing dependencies to other binding site positions share the disadvantage that the number of parameters increases exponentially with the number of positions considered. This problem is addressed by variable order Bayesian networks [35], variable length permuted Markov models [36], and hybrid-order models [37], which locally adapt the number of positions considered.

In principle, all of these models could also be employed for the prediction of TAL effector target sites, where a direct application would require to learn distinct parameters for the target sites of each TAL effector. However, the number of validated target sites of individual TAL effector is currently not sufficient to reliably estimate the parameters of any of these models. More importantly, such an approach would render the target site prediction for TAL effectors with currently unknown targets impossible. The ab-initio prediction of zinc finger transcription factor binding sites poses similar problems, which are addressed by an approach Kaplan *et al.* [38] specifically designed for that class of transcription factors. Regarding TAL effectors, this issue is addressed by several approaches specifically designed for the prediction of TAL effector target sites, which are outlined in the following.

We give an overview of current tools for the prediction of TAL effector target sites and TAL effector nuclease target sites in Table 1. *Target Finder* of the TALE-NT 2.0 suite [18], [39] predicts target sites of a TAL effector based on its RVD sequence. To this end, Target Finder represents RVD-dependent binding specificities as probabilities for the individual nucleotides, which are hard-wired into the code. These probabilities are combined as columns of a TAL effector-specific position weight matrix (PWM) model, which is used to scan user-supplied input sequences, promoteromes, or genomes for putative target sites. At the 5′ end of the target site, the user may either choose to restrict predictions to those with a preceding T, or to allow nucleotide C. Target Finder is available as a web-server and a stand-alone command line application, where the latter is published under an open-source license.

**Table 1:** Overview of tools for predicting TAL effector (TALE) and TAL effector nuclease (TALEN) target sites.

	TALgetter	Target Finder	Storyteller	TALVEZ	Paired Target Finder	idTALE
Prediction of TALE target sites	yes	yes	yes	yes	no	no
Prediction of TALEN target sites	no	no	no	no	yes	yes
Web server	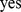 a	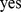 b	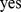 c	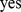 d	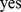 e	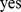 f
Custom input limit (web-server)	100 mb	5 kb	unkown	unknown	5 kb	N/A^g^
Stand-alone application	yes	yes	yes	yes	yes	no
Local web server	yes	no	no	no	no	no
Access	free	free	on e-mail request	on e-mail request	free	free
Method published	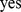 h	yes [39]	no	no	yes [39]	no
Method/Model	local mixture model	modular PWM	unknown	unknown	modular PWM	unknown
Adaptable to new data	yes	in source code	unknown	unknown	in source code	no
Open Source License	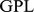 i	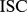 j	no	no	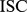 j	no

a http://galaxy.informatik.uni-halle.de.

b https://tale-nt.cac.cornell.edu/node/add/talef-off.

c http://bioinfo-prod.mpl.ird.fr/xantho/tales/.

d http://bioinfo.mpl.ird.fr/cgi-bin/talvez/talvez.cgi.

e https://tale-nt.cac.cornell.edu/node/add/talef-off-paired.

f http://idtale.kaust.edu.sa.

g only pre-defined data sets;

h this manuscript;

i GNU General public license;

j Internet Systems Consortium license, only stand-alone application;

Storyteller and TALVEZ are provided as a web-server and stand-alone application as well. However, the methods behind both approaches are not published, yet, and are accessible only on e-mail request. For these reasons, we do not consider Storyteller and TALVEZ in the remainder of this paper. Paired Target Finder and idTALE use RVD-dependent binding specificities to predict target sites of TAL effector nucleases, which function as homo- or hetero-dimers to specifically cut genomic DNA. While Paired Target Finder is available as a web-server and command line application, idTALE is only available as a web-server and can only be applied to pre-defined input data sets. Both approaches are applicable to TAL effector nucleases but not to TAL effectors.

In this paper, we propose a new statistical model for the prediction of TAL effector target sites, which represents *importance* of RVDs and their *binding specificity* independently. The concept of importance is related to the *efficiency* of RVDs reported by Streubel *et al.* [6]. However, while efficiency denotes the *positive* contribution of specific RVDs to the transcriptional activation by TAL effectors, importance additionally affects the penalty for non-matching nucleotides in a target site. We model the importance of RVDs by a binary hidden variable that represents interaction or non-interaction of an RVD with the corresponding nucleotide. Important RVDs are assumed to interact with the DNA in the majority of cases and, hence, should obtain a high probability of interaction. In case of interaction of RVD and nucleotide, binding specificities are represented by probabilities for the interacting nucleotides that depend on the corresponding RVD. If an RVD does not interact with the DNA, the probabilities of nucleotides are determined by the genomic context. In the proposed model, neither the importance nor binding specificity of an RVD depends on the position of the repeat or on other RVDs in the TAL effector. These assumptions allow for a model with an acceptable number of parameters, which is independent of the number of repeats in a TAL effector. In contrast to previous approaches, the parameters of the proposed model are computationally estimated from known pairs of TAL effectors and target sites. This allows for a rapid and automatic adaption of the model parameters as new target sites are validated. We call the tool using this new approach TALgetter – *TAL* effector tar*get* si*t*e find*er*. TALgetter is implemented within the open-source Java library Jstacs [40], and will be part of the next public release. A web-application of TALgetter is available at http://galaxy.informatik.uni-halle.de, and can also be installed in a local Galaxy [41]–[43] server. In addition, we provide a command line version of TALgetter at http://jstacs.de/index.php/TALgetter. Both, the web-application and the command line application, also allow a user to estimate new model parameters from custom training data. Hence, users can adapt the parameters of the TALgetter model to improved sets of validated TAL effector target sites, which are to be expected in the near future.

The mechanism of transcriptional activation by TAL effectors is still not fully understood. However, there are indications that the presence of a suitable TAL effector target site in a promoter is not always sufficient to induce transcription of the downstream gene [3], [9], [44], [45]. Likely, other factors, e.g., promoter elements surrounding the target site, are required for efficient transcriptional activation, too. Since these factors are yet unknown, they cannot be incorporated into a computational model. Hence, we propose to currently assist the search for functional target sites by experimental approaches to measure activation. In this paper, we use gene expression microarray data of *Oryza sativa* (rice) and *Citrus sinensis* (sweet orange) measured after infection with different *Xanthomonas* strains, and gene expression microarray data of transgenic *Arabidopsis thaliana* lines endogenously expressing a TAL effector for this purpose.

The remainder of this paper is structured as follows. In the section *Materials and Methods*, we define the proposed statistical model, and introduce public and in-house gene expression microarray data sets, as well as sequence data used in our studies. The *Results* section is split in several parts. First, we investigate the capability of our approach to predict known target sites of different TAL effectors in rice. In a second part, we compare the prediction accuracy of TALgetter to the Target Finder of TALE-NT based on gene expression microarray data. In a third part, we scrutinize the RVD binding specificities and importances estimated for the proposed model. We then investigate properties of TAL effector target sites, namely positional preference and the relationship to core promoter elements, revealing novel insights into the biology of TAL effector target sites. Finally, we predict several new putative TAL effector target sites in *Oryza sativa* and *Citrus sinensis*, which are supported by gene expression data.

## Materials and Methods

In this section, we define the statistical model used by TALgetter, we describe how the parameters of this statistical model are estimated from training data, and we explain how the trained model is used to scan genomes, promoteromes or other input sequences for putative target sites. Subsequently, we describe the gene expression data obtained from microarray experiments and sequence data used in the studies of this paper.

### Model

The statistical model employed by TALgetter is defined by its likelihood, which is derived in the following. Let 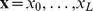 be an input DNA sequence of length 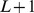, where 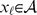 represents the nucleotide at position 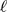 of the sequence, and 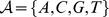. Let 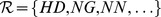 be the alphabet of known RVDs, and let 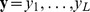 with 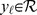 be the RVD sequence of the TAL effector of interest. For each position 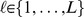, we model the potential interaction of nucleotide 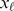 and RVD 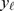, while nucleotide 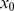 directly preceding the interacting positions is modelled independently of the RVD sequence.

We can decompose the likelihood 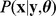 of input sequence 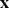 given the sequence of RVDs **y** and model parameters 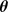 as
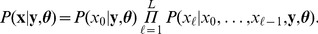
(1)where 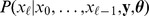 is the probability of nucleotide 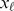 given all previous nucleotides, the complete RVD sequence 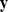, and all model parameters 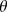.

Since a strong preference for nucleotide T at position 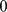 directly preceding the target site at the 5′ end has been observed [2], while nucleotides C and A are accepted in natural targets as well [9], [46], this position is included in the model. We assume that the nucleotide preference at position 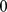 does not depend on the RVD sequence or the specific TAL effector and, hence, define
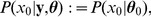
(2)where 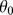 denotes the parameters of the model at position 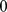.

As motivated in the introduction, we model binding specificity and importance of an RVD independently. In addition, we impose several independence assumptions:

1. If an RVD of a repeat of the TAL-effector interacts with the DNA, the probability of nucleotide 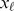 to occur at position 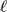 of the DNA only depends on the RVD 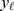.
2. If that RVD does not interact with the DNA, the probability of nucleotide 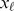 only depends on the genomic *context*. We define the context as the previous 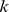 nucleotides 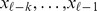.
3. The binding specificity of an RVD is independent of the position within the target site and independent of the other RVDs of the TAL effector.
4. The importance of an RVD is independent of the position of its repeat within the TAL effector and independent of the other RVDs of the TAL effector.

These assumptions may be formulated as a *local mixture model* for each position 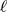 reflecting interaction vs. non-interaction of RVD and DNA by a binary hidden variable 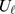 with values 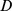 and 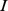, respectively. Let 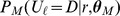 be the probability that RVD 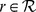 interacts with the DNA, and let 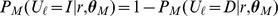 be the converse probability that no interaction occurs. Finally, let 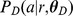 be the probability of nucleotide 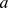 given an interaction of RVD 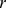 with the DNA at the corresponding position, and let 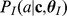, 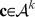 be the probability of nucleotide 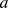 given its context 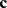 under the condition that no interaction occurs. The context 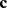 does not extend beyond the 5′ end of the sequence 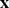, and, hence, the context considered may be shortened. With these definitions, we have
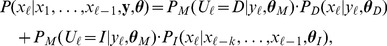
(3)where 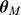 denotes the parameters of the mixture probabilities, 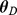 denotes the parameters of the RVD-dependent component, and 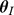 denotes the parameters of the RVD-independent component, and 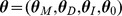. For all subsequent studies, the order 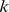 is fixed to 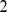.

#### Parameter estimation

Given an input data set 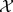 of 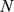 independent pairs 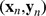 of target site sequence and RVD sequence of the corresponding TAL effector, the likelihood of this data set is given as
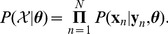
(4)


One commonly used principle for estimating the parameters of statistical models is the generative maximum likelihood principle. However, two reasons suggest employing the Bayesian maximum a-posteriori (MAP) learning principle, which additionally imposes a prior on the model parameters. First, we have prior knowledge about binding specificities and importance of binding that are not fully covered by our training data. Second, our training data contains only a very limited number of TAL effectors and corresponding target sites. For this reason, we cannot be certain that binding events which are not observed in the training data can never occur in functional target sites. More severely, some rare but known RVDs are not present in any TAL effector of our training data. Nonetheless, the model should be able to assign likelihoods to putative target sites of TAL effectors containing such rare RVDs.

We use independent Dirichlet priors on each sub-set of parameters defined on a common simplex, which results in a product-Dirichlet prior on the full set of parameters. We denote the prior on the parameters 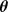 given hyper-parameters α as 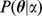. Details on the prior and its hyper-parameters can be found in supplementary Text 16. The posterior probability 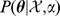 of the parameters given data and hyper-parameters is proportional to the product of likelihood and prior, i.e.,
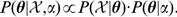
(5)


We estimate the parameters 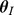 of the RVD-independent component from all annotated promoter sequences of *A. thaliana* and *O. sativa* by the Bayesian maximum a-posteriori principle, and fix these parameters before estimating the parameters of the remaining components.

We estimate the optimal parameters 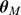, 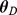, and 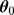 of the remaining components by the Bayesian maximum a-posteriori principle as well and obtain
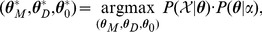
(6)which is optimized numerically by a second-order quasi-Newton method on the data set 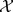 of 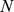 pairs 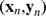 of target and RVD sequence. For unconstrained numerical optimization, we transform the parameters to the natural parameterization [47], [48].

The training data set used in this study comprises known pairs of TAL effector and target site from [2], [3], [6], [7], [9], [10], [13], [20], [45], [49]. Besides natural target sites, this set also contains artificial target sites from mutation experiments, which are often highly similar within one series of experiments. To avoid a bias towards such artificial sites, we downweight these such that all target sites from one series of mutation experiments obtain the same weight as one natural target site. If quantitative data, for instance GUS activity, are available, these are used to split the weights within a series of mutation experiments. All pairs of target sites and corresponding weights are listed in supplementary Table S1 and are also available in the format required by TALgetter as supplementary Data S1.

The values of all parameters of the TALgetter model as estimated from these training data are listed in supplementary Text S1, and we provide a graphical representation in the section *Binding specificities and importance of RVDs*.

#### Predicting target sites

Once the parameters have been estimated, we predict target sites of a given TAL effector by scanning input sequences, for instance the promoterome of an organism, using the trained model. Given the RVD sequence of length 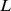 of the TAL effector of interest, we use a sliding window of length 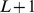 to extract sub-sequences of this length. Each of these sub-sequences serves as an input to the trained model and the likelihood given the TAL effector sequence is computed according to equations (1) and (3). Subsequently, sub-sequences are ranked according to their likelihood values.

Since the absolute value of the likelihood directly depends on the length of the RVD sequence, and, hence, on the width of the sliding window used for scanning, the obtained likelihood values are in general not comparable between different TAL effectors. To overcome this issue, we additionally compute empirical p-values for the putative target sites based on their likelihood values. To this end, we generate a data base of random sequences of at least the total length of all scanned input sequences. We generate these random data by drawing sequences from a homogeneous Markov model of order 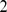 trained on the input sequences. For these random data, we compute the likelihoods of sub-sequences in the same manner as for the actual input sequences. We then compute the p-value of a putative target site by determining the percentile corresponding to the observed likelihood value on the distribution of likelihoods obtained for the random data base.

### Data

#### Public *Oryza sativa* gene expression microarrays

For assessment and predictions in *Oryza sativa* (rice), we use publicly available gene expression data from PLEXdb [50]. Of the experiments in the data base, we choose those that are obtained i) for *Oryza sativa* ssp *japonica* cv Nipponbare, ii) after infection with a *Xanthomonas* strain expressing at least one known TAL effector, and iii) 24 h post infection (hpi). We restrict our studies to *Oryza sativa* ssp *japonica*, because a well curated and annotated genome is only available for *japonica* but not for *indica* rice.

For finding differentially expressed genes after infection with *Xanthomonas*, we download the expression intensities obtained after RMA [51] normalization. The resulting experiments are listed in Table 2. The TAL effectors expressed by the *Xanthomonas* strains which are used in the studies and their RVD sequences are listed in supplementary Text S1.

**Table 2:** *Oryza sativa* ssp *japonica* microarray experiments from PLEXdb used in this paper.

PLEXdb ID	GEO ID	*Xanthomonas* 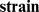 a	Experiments	#arrays
OS3	GSE16793	*Xoo* 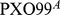 , *Xoc* BSL256	24 hpi, mock	12
OS38	GSE19844	*Xoo* BAI3, *Xoo* BAI3 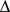 *talC*	24 hpi, mock	9
OS66	GSE19844	*Xoo* 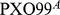 , *Xoo* PXO86, *Xoo* MAFF311018, *Xoo* 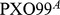 ME1 ( 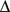 *pthXo6*, *avrXa27*), *Xoo* 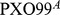 ME2 ( 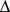 *pthXo1*)	24 hpi, mock	27

a *Xoo*: *X. oryzae* pv. *oryzae*; *Xoc*: *X. oryzae* pv. *oryzicola*.

Depending on the experiment, we extract different lists of differentially expressed genes based on the log-fold change of a target data set versus a control data set. Specifically, target and control data sets are *infected* vs. *mock* or *wildtype* vs. *mutant*. All considered pairs of data sets are listed in Table 3. In cases where we compare a wildtype strain to a mock experiment, we expect to find – besides others – target genes of the TAL effectors expressed by the wildtype strain to be up-regulated in the gene expression data of the microarray studies. If we compare wildtype strains to single or double mutants, we expect to find as differentially expressed the target genes of those TAL effectors that are not functional in the mutant.

**Table 3:** Data sets of putative target genes of expressed TAL effectors obtained from comparative studies (in alphabetical order).

Name	PLEXdb ID	Target	Control
BAI3	OS38	*Xoo* BAI3, 24 hpi	*Xoo* BAI3 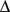 *talC*, 24 hpi
MAFF311018	OS66	*Xoo* MAFF311018, 24 hpi	mock
PXO86	OS66	*Xoo* PXO86, 24 hpi	mock
PXO99	OS66	*Xoo* 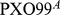 , 24 hpi	mock
PXO99AME1	OS66	*Xoo* 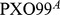 , 24 hpi	*Xoo* 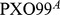 ME1, 24 hpi
PXO99AME2	OS66	*Xoo* 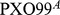 , 24 hpi	*Xoo* 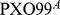 ME2, 24 hpi
XOC	OS3	*Xoc* BSL256, 24 hpi	mock
XOO	OS3	*Xoo* 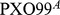 , 24 hpi	mock

Although these gene expression data are a valuable source of information about *in vivo* effects of *Xanthomonas* infections, they also entail drawbacks for a systematic evaluation of TAL effector target site predictions. First, the observed changes in expression levels may be the result of a mix of the effects of different TAL effectors and we cannot distinguish, which of the TAL effectors expressed by a *Xanthomonas* strain is responsible for a change in the expression of a gene. Second, the observed expression levels of plant genes are not only directly influenced by TAL effectors but may be also affected by secondary effects due to other type III effectors secreted simultaneously into the plant cell, or by general plant responses due to the infection.

For this reason, we additionally design a more controlled environment for studying the effects of a single TAL effector as described in the following.

#### *Arabidopsis thaliana* gene expression microarrays

We generate transgenic *Arabidopsis thaliana* plants to study the effect of individual TAL effectors on plant gene expression without secondary effects from the bacterial pathogen [2]. Three independent transgenic *A. thaliana* Col-0 lines are generated via *Agrobacterium*-mediated transformation that carry the *hax2* TAL effector gene from *Arabidopsis*-pathogenic *Xanthomonas campestris pv. campestris* under control of an ethanol-inducible promoter [2], [52]. Generation of three independent lines should compensate for changes in individual gene expression patterns due to the insertion of the T-DNAs. We sample leaf tissues of transgenic and non-transgenic plants from segregating T2 populations 24 hours post ethanol treatment. Because expression of *hax2* in *A. thaliana* Col-0 results in purple-colored leaves due to anthocyanin accumulation, the presence or absence of the *hax2* transgene can be determined phenotypically and is verified by PCR analysis [2]. We treat both, transgenic and non-transgenic plants, with ethanol to compensate for any general effect of ethanol on *A. thaliana* gene expression. The expression levels of *A. thaliana* genes are measured by Affymetrix Ath1 microarrays (imaGenes GmbH, Berlin, Germany), RMA normalized, and fold changes are determined for each of the 3 transgenic lines compared to 3 non-transgenic lines independently. The final list of differentially expressed genes is then determined as the intersection of the lists obtained for the three independent experiments for a given threshold on the log-fold change.

#### Public *Citrus sinensis* gene expression microarrays

In a third study, we predict putative target sites of TAL effectors of *Xanthomonas axonopodis pv. citri* (*Xac*) in *Citrus sinensis*. The RVD sequences of the TAL effectors considered are listed in supplementary Text S1.

We obtain gene expression data of *C. sinensis* of plants infected by Xac and mock plants 48 h post infection from PLEXdb (PLEXdb ID: CT2). As for the rice microarrays, we download expression data normalized by RMA and compute average log-fold changes of infected (2 replicates) versus mock (2 replicates) plants.

#### Sequence data

We obtain *O. sativa* pseudomolecules and gene annotations from the MSU Rice Genome Annotation Project [53] at ftp://ftp.plantbiology.msu.edu/pub/data/Eukaryotic_Projects/o_sativa/annotation_dbs/pseudomolecules/version_7.0/all.dir/all.chrs.con and ftp://ftp.plantbiology.msu.edu/pub/data/Eukaryotic_Projects/o_sativa/annotation_dbs/pseudomolecules/version_7.0/all.dir/all.gff3, respectively. For the benchmark studies, we extract 1000 bp upstream of the start codon for all annotated genes including splicing variants. For some of the biological studies, we additionally extract sequences extending from 1000 bp upstream of the annotated transcription start site to the position directly preceding the start codon.

We obtain *A. thaliana* upstream sequences relative to the start codon from TAIR [54]. TAIR also provides updated gene annotations of the probe sets of the Affymetrix Ath1 chip for the TAIR10 gene annotations, which we use throughout our studies for consistency.

We obtain *C. sinensis* scaffolds and gene annotations from ftp://ftp.jgi-psf.org/pub/JGI_data/phytozome/v6.0/Csinensis. These sequence data were produced by the US Department of Energy Joint Genome Institute http://www.jgi.doe.gov/ in collaboration with the user community. For all annotated genes, we extract 1000 bp upstream of the start codon for predicting target sites. In addition, we extract the corresponding transcripts and establish gene annotations for the microarray probe sets by blasting [55] the probe sequences to these transcripts, where we allow at most two mismatches between probe and target sequence.

## Results/Discussion

### Recovering known target sites

In this section, we examine known TAL effector target sites in *O. sativa*, and analyze if these target sites are recovered by TALgetter. To this end, we consider two settings. In the first setting, we consider each TAL effector in turn and exclude all TAL effectors with RVD sequences identical to the TAL effector considered from the training set in a cross validation-like manner. For testing, we scan the standard region of 1 kb upstream of the start codon of all rice genes and rank the predictions of TALgetter according to the corresponding likelihood. We refer to this setting as *TALgetter CV*.

In the second setting, we use the final version of TALgetter, where we use the complete training data. This version is available as a web-application and command line program. For testing, we scan regions from 300 bp upstream the transcription start site (TSS) to 200 bp downstream the TSS or the start codon, whichever comes first. This choice will be motivated in the section *Positional preference of target sites*. We refer to this setting as *TALgetter final*.

The ranks of the known TAL effector target sites achieved in these two settings are listed in Table 4. For TALgetter CV, we find the known target sites of Tal1c [3], PthoXo6 [2], PthXo7 [2], and TalC [9] among the top 10 promoterome-wide predictions of TALgetter, while the known target site of PthXo1 [2], [3] is predicted at rank 20. For TALgetter final, we find the known target sites of all these TAL effectors at rank 1 or 2.

**Table 4:** Ranks of known TAL effector target sites.

TAL effector	Target gene	Locus ID	Reference	TALgetter CV	TALgetter final
Tal1c/XOCORF_0460	OsHEN1	Os07g06970	[3]	1	1
PthXo6	OsTFX1	Os09g29820	[2]	8	2
PthXo7	OsTFIIa 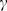 1	Os01g73890	[2]	3	2
TalC	Os11N3	Os11g31190	[9]	2	2
PthXo1	OS8N3	Os08g42350	[2], [3]	20	1
PthXo3	Os11N3	Os11g31190	[49]	479	240
AvrXa7	Os11N3	Os11g31190	[7]	732	324

Ranks of known TAL effector target sites among the TALgetter predictions in independent cross validation-like experiments (TALgetter CV) and for the final version used in the web-application and command line program (TALgetter final).

For the known target sites of PthXo3 and AvrXa7, the achieved ranks in both settings are considerably worse. Interestingly, these two TAL effectors contain atypical, long repeats, which might influence the overall binding of the TAL effector. Since such atypical repeats are not specifically modelled by TALgetter, this might explain the high ranks of the true target sites of PthXo3 and AvrXa7. However, once the impact of long repeats on TAL effector binding are understood, these could be implemented in the modular structure of TALgetter. Notably, the same effect can also be observed for Target Finder, where the known target sites of PthXo3 and AvrXa7 obtain ranks 558 and 1543, respectively. We compare the prediction accuracy of TALgetter and Target Finder in more detail in the next section.

### Comparison to Target Finder

In the first part of this section, we consider public gene expression microarray data of *O. sativa* studying the effects of *Xanthomonas* infections on the transcriptome. Using these gene expression data, we determine sets of genes that are up-regulated upon *Xanthomonas* infection. Under the assumption that a considerable subset of these genes is directly up-regulated by TAL effectors, we compare the number of predicted target sites of TALgetter and Target Finder that are consistent with the observed up-regulation. Besides virulence targets, these up-regulated genes presumably also include collaterally induced genes. When predicting target sites with TALgetter, we use the TALgetter CV setting described in the previous section and exclude all TAL effectors with RVD sequences identical to the current one from the training set. For Target Finder, we use the publicly available version (https://boglab.plp.iastate.edu/node/add/talef-off, https://github.com/njbooher/boglab_talesf) having fixed binding specificities, which might include knowledge from known target sites of a TAL effector considered. In the second part, we repeat this analysis for genes that are up-regulated in *A. thaliana* plants that endogenously express the TAL effector Hax2.

#### Comparison on public *O. sativa* gene expression microarrays

We compare the prediction accuracy of TALgetter to that of Target Finder of the TALE-NT suite using the public gene expression microarray data of *O. sativa* described in section *Data*. To this end, we consider for each experiment listed in Table 3 those genes that are at least two-fold up-regulated 24 h post infection, corresponding to a threshold of 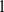 on the log-fold changes.

For each of the experiments, we obtain the RVD sequences of the TAL effectors expressed by the *Xanthomonas* strains used in the infection (cf. supplementary Text S1). We then use both tools, TALgetter and Target Finder, to predict target sites of these TAL effectors in the sequences 1 kb upstream of the start codon of all annotated *O. sativa* genes, which is also the standard for promoterome-wide scans in the Target Finder web-application.

For the following comparisons, we only consider those genes that are represented by at least one probe set on the Rice 57k microarray, since we have no knowledge about the regulation of genes that are not on the chip. For Target Finder, we basically use the default parameters with two exceptions: First, we switch off the option to scan the reverse complementary sequence, because natural TAL effector act as transcriptional activators only if the activation domain is oriented towards the downstream gene. Second, we examine the variant of Target Finder filtering for a T at position 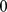, and the variant additionally allowing for a C at position 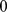 independently.

In Target Finder, better predictions are assigned lower scores, where the score is basically the negative log-likelihood obtained from the position weight matrix model. By default, Target Finder includes all putative target sites into its predictions that yield a score that is at most 3 times the best possible score for the current TAL effector. However, the number of predicted target sites varies greatly between different TAL effectors. For instance, Target Finder reports only 7 target sites for TAL effector XOO2160_MAFF, whereas more than 1.4 million target sites are predicted for XOCORF_1565 using the default threshold. In contrast, TALgetter predicts between 6 and 314 target sites for the TAL effectors considered using the default threshold of 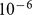 on the empirical p-values. Since the total number of *O. sativa* genes including splicing variants is only about 66 000, we decide to limit the number of predictions in the same manner for both tools: For each TAL effector, we limit the predictions of both tools to the top 10, 20, 50, and 100 predicted target sites, in the following also referred to as *rank cutoff*. We then compile the total predictions for a given experiment as the union of the predicted target sites for all TAL effectors relevant in this experiment. For instance, Xoo 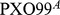 expresses 19 known TAL effectors, of which 16 are functional [56]. If we limit the predictions to the top 20 target sites for each TAL effector of *Xoo* 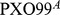, we obtain a total number of 320 predicted target sites. The number of corresponding target genes is at most 320, since in rare cases multiple target sites are predicted in the promoter of one gene. For *Xoo* 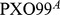 and a rank cutoff of 20, we obtain 276, 276, and 273 different target genes for TALgetter, Target Finder filtering for a T, and Target Finder filtering for a T or a C at position 0, respectively.

We use as a measure of accuracy the number of genes with predicted target sites for a specific rank cutoff that are also up-regulated according to the microarray experiment. Since the number of up-regulated genes only depends on the threshold on the log-fold change in the given experiment, this number is proportional to the recall. Since the number of predicted target genes is limited by the rank cutoff and almost equal for the different tools, it is also roughly proportional to the precision. Hence, we consider it a suitable measure of the overall performance of a prediction tool.

**Figure 1:**
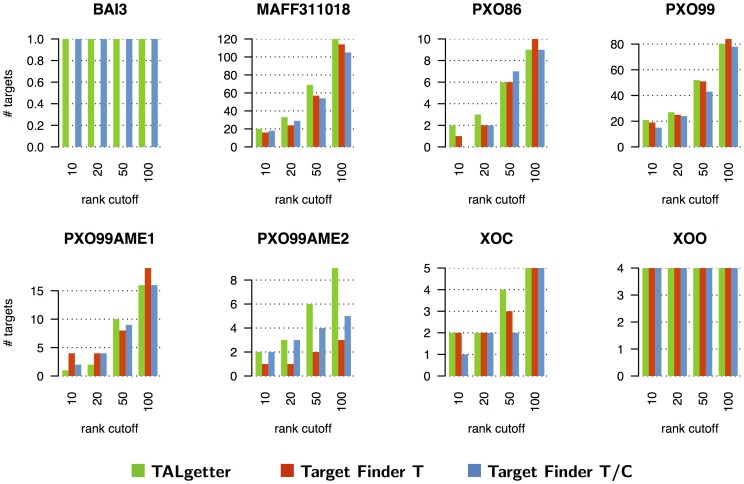
Comparison of TALgetter to Target Finder with a T (Target Finder T), or both T and C (Target Finder T/C) at position 0 on public gene expression data. We consider as performance measure the number of predicted targets that are supported by up-regulation according to gene expression data after *Xanthomonas* infection using a log fold-change of 1. Performance is measured for different rank cutoffs (Top 10, 20, 50, and 100 predictions) on the predictions for each TAL effector.

**Figure 2:**
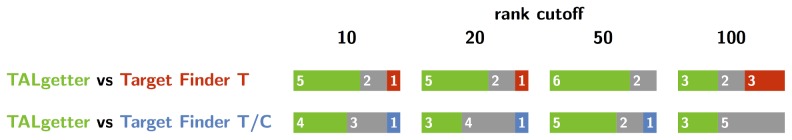
Summary of the evaluations presented in Figure 1. For each rank cutoff (10, 20, 50, 100), we count the number of data sets where a prediction program outperforms the other (bars colored identical to program), or both score equally well (bars colored gray).

The results of this evaluation procedure are presented in Figure 1. In addition, we give a summary of the evaluations in Figure 2.

First, we compare the prediction accuracy of TALgetter to that of Target Finder filtering for a T at position 0 (Target Finder T) using a rank cutoff of 10. We find that for the data sets BAI3, MAFF311018, PXO86, PXO99, and PXO99AME2, TALgetter (green bars) predicts more target sites that are present in promoters of genes which are also transcriptionally induced in the corresponding microarray studies than Target Finder T (red bars). For two experiments, namely XOC and XOO, the number of recovered genes is equal for both tools. Finally, Target Finder T finds more up-regulated genes than TALgetter for the data set PXO99AME1. We find the corresponding aggregate values in the first column of Figure 2. TALgetter yields a larger number of recovered genes for 5 data sets, the opposite is true for one data set, and both tools yield an equal performance in two cases.

This picture is similar for the comparison to Target Finder filtering for a T or a C at position 0 (Target Finder T/C) using the same rank cutoff. For 4 data sets, namely MAFF311018, PXO86, PXO99, and XOC, TALgetter recovers more up-regulated genes than Target Finder T/C. We observe the opposite only for the data set PXOAME1, while for the remaining three data sets both tools score equally well.

As can be observed from Figures 1 and 2, this picture is widely consistent for rank cutoffs 20 and 50, where TALgetter predicts a greater number of up-regulated genes than Target Finder T in 5 and 6 cases, and a greater number of up-regulated genes than Target Finder T/C for 3 and 5 data sets, respectively.

For a rank cutoff of 100, which for instance already results in a total number of 1600 predictions considered for the 16 TAL effectors of the data set PXO99, TALgetter and Target Finder T achieve a comparable number of up-regulated genes. In contrast, Target Finder T/C still finds less up-regulated genes than TALgetter for 3 out of the 8 data sets, whereas the opposite is true for none of the data sets.

Summarizing the results on all data sets considered, we may state that TALgetter shows a slightly improved overall prediction performance compared to Target Finder using the number of predicted targets consistent with up-regulation after *Xanthomonas* infection as performance measure. This is especially the case for lower rank cutoffs. We consider this case of the greatest practical relevance, since often a user needs to scan lists of predictions by eye, for instance to find putative candidates for experimental validation.

Since the results of the comparison might be specific to the threshold on the fold-changes chosen to determine up-regulated genes, we repeat this analysis for a threshold of 0.5 on the log-fold change. The outcome of this evaluation is presented in Figure S1 and S2 in analogy to Figure 1 and 2, respectively. Although the set of genes considered as up-regulated may be contaminated to a larger extent by false positives for such a low threshold, we find that TALgetter still performs better than both variants of Target Finder. However, the differences between the tools are less pronounced for a threshold of 0.5.

**Figure 3:**
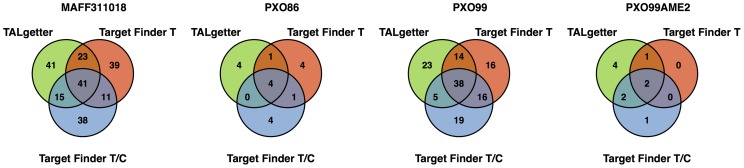
Venn diagrams of the predictions of the three programs using a log fold-change of 1 and a rank cutoff of 100.

The relevance of a novel approach for TAL effector target site prediction also depends on the number of additional targets that we gain compared to previous approaches. Therefore, we investigate if the predictions of TALgetter and Target Finder are largely overlapping or if both approaches rather predict complementary target sites. We consider the overlap of predicted target genes between TALgetter and the two variants of Target Finder in Figure 3 for 4 representative data sets, while the Venn diagrams for all data sets are given in supplementary Figure S3. As a general tendency, we find that the number of target genes that are uniquely predicted by one of the three tools is surprisingly large compared to the number of target genes that are consistently predicted by all three approaches. On first sight, this is especially surprising for the overlap between the two TALgetter variants. However, it can be explained by the different filter on position 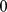 combined with a fixed rank cutoff, because, on average, we expect only half of the predictions of Target Finder T/C to have a T at that position.

Notably, the Venn diagrams reflect that TALgetter on the one hand accepts any nucleotide at position 0, resulting in an exclusive overlap with Target Finder T/C, but on the other hand learned a strong preference for nucleotide T at that position (cf. section *Binding specificities and importance of RVDs*) leading to a greater overlap with Target Finder T.

For all data sets, the number of target genes exclusively recovered by TALgetter is equal to or greater than the number of exclusive targets of each of the Target Finder variants. For instance, TALgetter predicts 41 additional putative TAL effector targets for the data set MAFF311018 that would not have been predicted by any of the Target Finder variants. In case of PXO99AME2, TALgetter exclusively predicts 4 target genes, whereas only 1 target gene is predicted by Target Finder but not by TALgetter. From an experimental perspective, these exclusively predicted targets underline the scientific value of TALgetter, since these may include virulence targets that would have been missed using existing approaches.

#### Comparison on *A. thaliana* gene expression microarrays

As an independent validation data set with a reduced number of side-effects of the infection, we consider the microarray experiment of *hax2*-transgenic *A. thaliana* plants introduced in section *Data*.

**Figure 4:**
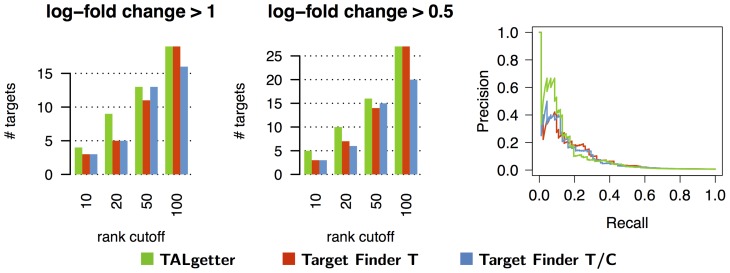
Comparison of TALgetter to Target Finder on the gene expression data from transgenic *A. thaliana* lines. We consider as performance measures the number of predicted targets consistent with up-regulation in gene expression microarrays for different rank cutoffs and thresholds of 1 and 0.5 on the log-fold changes (left panel), and the precision-recall (PR) curve (right panel) using a threshold of 1 on the log-fold changes.

The results of the evaluations on this data set are presented in Figure 4. In the left panel of Figure 4, we again consider the number of target genes recovered by TALgetter and the two Target Finder variants for different rank cutoffs and thresholds of 1 and 0.5 on the log-fold changes. We find that TALgetter yields an improved prediction performance compared to the Target Finder variants for most rank cutoffs considered, whereas the opposite is never the case.

In contrast to the previous microarray data for *O. sativa*, the controlled environment of transgenic plants endogenously expressing a single TAL effector gene (*hax2*) allows us to distinguish between target and non-target genes more reliably, since general effects due to other translocated *Xanthomonas* effectors or a general plant response to the infection are eliminated. In addition, the expression of a single TAL effector allows us to attribute transcriptional activation to a specific TAL effector.

Hence, we additionally plot a precision-recall curve of the predictions of the three tools. To this end, we classify all genes with a log-fold change greater than 1 as targets and all genes with an absolute log-fold change of less than 0.5 as non-targets. We plot the precision-recall curves of TALgetter and the two Target Finder variants using a varying threshold on the prediction scores in the right panel of Figure 4. We find that for recalls above 0.2, none of the tools yields an acceptable precision. For a recall of approximately 0, all tools yield a single correct prediction leading to a precision of 1.0. For all other recalls, TALgetter is the only approach that achieves precisions above 0.5 for some of the recall values, whereas more than half of the predictions of Target Finder are false positives. This result is an additional indication of the improved prediction accuracy of TALgetter compared to Target Finder.

Summarizing the comparison of TALgetter to Target Finder, the assessment of prediction accuracy presented in this section demonstrates that TALgetter yields an improved overall prediction performance compared to Target Finder. TALgetter uniquely predicts many targets that are not predicted by Target Finder, which diversifies the types of TAL effector target sites that can now be discovered by computational approaches. We focus on the binding specificities learned by TALgetter and the properties of target sites predicted by TALgetter in the following sections. However, depending on the goal of a study, it might also be worthwhile to use both, TALgetter and Target Finder, for predicting putative target sites and to combine the predictions of both approaches. For instance, the union of the predictions of both approaches might cover a broader range of target sites, since we observe a considerable number of unique predictions for all approaches.

### Binding specificities and importance of RVDs

**Figure 5:**
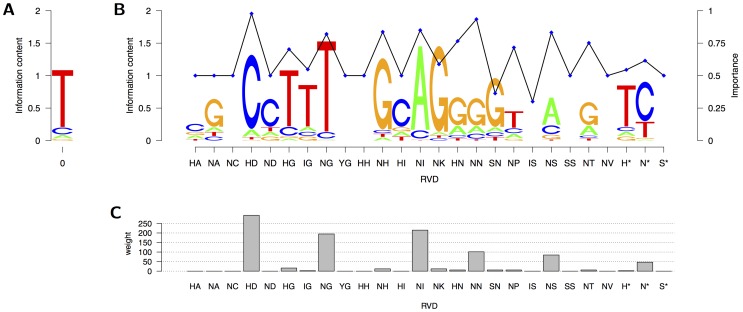
Visualization of the parameters of TALgetter. (A) The nucleotide preferences for position 0 are visualized as a sequence logo. (B) The binding specificities for the different RVDs are plotted in analogy to sequence logos as well, whereas the probabilities 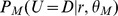 representing importance of RVDs are plotted against the RVDs as blue points connected by a black line. (C) Total weight of each RVD in the training data set.

We visualize the binding specificities and importances of the different RVDs in Figure 5. Considering the nucleotide preferences at position 0 shown in panel (A) of Figure 5, we find that the most frequent nucleotide with a probability of 0.829 is T, followed by C with a probability of 0.100, A with a probability of 0.049, and G with a probability of 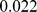, which is in accordance with previous findings [2], [3], [7], [8].

Turning to the binding specificities of RVDs, we find the highest specificities for HD (C), NG (T), NH (G), NI (A), and NK (G). This is in accordance to the experimentally determined DNA-specificities of these RVDs [2], [6], [13], [21], [57].

For HD, NG, NH, and NI, we also find a high importance. Hence, these RVDs are highly specific and mismatches according to the binding specificities are hardly tolerated. Other RVDs with a notably high importance are HN, NN, NP, NS, and NT, although these RVDs are less specific than the other high-importance RVDs.

As noted in the introduction, the concept of importance of RVDs is related but not identical to the efficiencies of RVDs as proposed by Streubel *et al.* [6], which classifies RVDs as *strong*, *intermediate*, and *weak*, respectively. TAL effectors with exclusively weak RVDs can not activate transcription of downstream genes, even if all binding specificities are matched [6]. Inclusion of three or more strong RVDs renders the TAL effectors fully functional. In contrast, more intermediate RVDs (e.g. six) are needed for full activity. The different efficiencies of RVDs likely reflect different DNA-binding strength and thereby affect overall TAL effector affinity to DNA.

The two RVDs classified as strong, namely HD and NN, also receive the highest importance in the TALgetter model. The intermediate RVDs NS and NH are assigned a fairly high importance as well, whereas the remaining intermediate RVDs, namely NP, HN, and NT, receive a lower importance. The RVDs NG and NI are assigned an importance comparable to that of the intermediate RVD NP, although these RVDs are classified as weak according to their efficiency. This result may be an effect of the related but different concepts of efficiency and importance, which we discuss in the following. An RVD with a low efficiency might prevent transcriptional activation in general, whereas a low importance has the effect that the binding specificities modeled by TALgetter for this RVD have a reduced influence on the overall score. An RVD with high efficiency has a strong positive influence on the transcriptional activation, whereas the contribution of an RVD with a high importance to the total score highly depends on the specificity. Hence, importance affects the penalty that is imposed if the binding specificity is not fulfilled, i.e., a nucleotide with a low probability for a specific RVD is present in a target site.

For some RVDs (HN, NN, NS, NT, N*), we observe a preference for more than one nucleotide, where we recognize gradually decreasing specificities. The most prominent example of this class of RVDs is N*, where we find a preference for C with a probability of 0.693, followed by T 0.272, and very low probabilities for A and G.

The RVD N* has experimentally been determined to specify for T and C with preference for T [6]. A preference for T is also expected, because RVDs are directly followed by a conserved glycine in the repeat sequence and, hence, N* might exhibit a binding preference similar to NG [5]. However, N* recognizes T and C in known TAL effector target sites instead [3], which differs from the RVD NG. In contrast, H* shows a binding preference that is similar to HG.

Figure 5 demonstrates that TALgetter correctly estimates the known specificities of RVDs [2], [3], [6], [13], [57]. Only amino acid 13 of each TAL effector repeat, i.e., the second amino acid of the RVD, interacts with the DNA base and should therefore be responsible for the RVD specificity [4], [5]. Accordingly, the parameters of TALgetter reflect that RVDs containing the same amino acid 13 have comparable specificities. Notable exceptions from this rule are the estimated binding preferences of HA and NA, and HI and NI, which can be explained by HA, NA, and HI being underrepresented in the training data set as shown in panel (C) of Figure 5.

For RVDs NC, YG, HH, IS, SS, NV, and S*, a uniform preference was estimated by TALgetter, since these RVDs are neither present in the training data nor do we have prior knowledge about their binding preference. However, under the assumption that only amino acid 13 of a repeat defines binding preference, we can overcome this issue by estimating a common binding preference for all RVDs with the same 13th amino acid. In this case, the probability 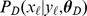 (cf. section *Materials and Methods*) does not depend on the full RVD 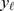, but only on its second amino acid. Due to the modular structure of the TALgetter model, we may still estimate an individual importance for each RVD. We refer to this variant of TALgetter as TALgetter13.

**Figure 6:**
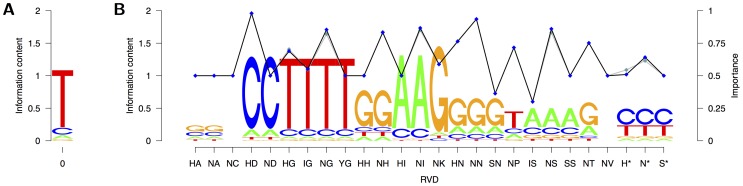
Visualization of the parameters of TALgetter with binding specificities determined by amino acid 13. (A) The nucleotide preferences for position 0 are visualized as a sequence logo. (B) The binding specificities given the different RVDs are plotted in analogy to sequence logos as well, whereas the probabilities 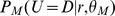 representing importance of RVDs are plotted against the RVDs as blue points connected by a black line. As a reference, we include the importance of RVDs using individual binding specificities as light blue points connected by a gray line.

The parameters estimated with these modifications are visualized in Figure 6. The binding preferences of the most prominent RVDs, namely HD, HG, NG, NI, NN, NS, and N* [5], remain highly similar to those estimated conditional on the complete RVDs (cf. Figure 5). By specification, the differences between HA and NA, and HI and NI are resolved, and the estimated binding preference is dominated by the more prominent RVD. For YG, HH, IS, SS, and S* that were assigned a uniform binding preference before, we gain binding preferences that are based on the preferences of the other RVDs with same amino acid 13. The importance of individual RVDs is only marginally affected by the modified binding preferences, with a slight decrease of binding importance for HG and H* and a slight increase for NG, NI, NS, and N*.

To investigate if the modified binding preferences influence the prediction accuracy of TALgetter13 compared to TALgetter, we repeat the assessment of prediction accuracy in complete analogy to the previous comparison to Target Finder. The results of this comparison are presented in supplementary Figure S4. We find that for 9 of the 32 combinations of rank cutoff and data set, TALgetter13 yields an improved prediction accuracy compared to TALgetter, whereas for 8 combinations the opposite is the case. For the remaining 15 combinations, both variants of TALgetter achieve an identical number of recovered target genes. Since we do not find an improved prediction accuracy for TALgetter13, we use TALgetter throughout the subsequent studies. However, TALgetter13 might be of value if we search for target sites of TAL effectors containing many rare RVDs. Hence, we include it as an option into the web-application and the command line program.

The variable importance of RVDs according to the parameters learned by TALgetter strengthens the observation that RVDs can differ in their efficiency and contribution to overall TAL effector function [6]. Our findings also suggest that the penalty of mismatching RVD-base combinations to overall TAL-binding differs for each individual RVD-base combinations, a concept that is novel.

### Positional preference of target sites

In the following, we investigate if TAL effector target sites are located in a preferred distance to either the start codon or the transcription start site (TSS) of target genes. To this end, we scan broader regions of upstream sequences, which span from 1 kb upstream of the transcription start site to the start codon as described in section *Data*, and collect the positions of the top 200 predicted target sites for each TAL effector studied. As in the previous comparison to Target Finder, we define as positives all genes that achieve a log-fold change greater than 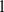 in the corresponding experiment. We additionally create a set of negatives by extracting all genes with an absolute log-fold change of less than 0.5. The sets of positive and negative genes are then combined with the target site positions collected from the predictions of TALgetter to obtain positive and negative sets of target site positions. In the following, we consider the unions of these sets across all microarray experiments for *O. sativa*.

**Figure 7:**
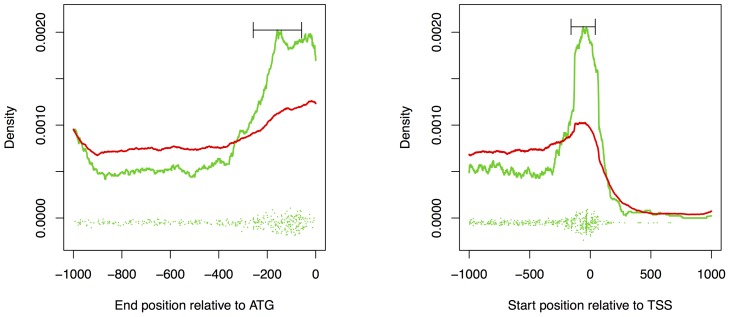
Positional preference of TAL effector target sites relative to the start codon (left) and the transcription start site (TSS, right). The estimated density of positions from the positive set is plotted as a green line, while the density of the negatives is plotted in red. The whiskers indicate the bandwith of the box kernel used to smooth the curves in a kernel density estimation. The green points at the bottom of the plots represent the distribution of positions from the positive set along the x-axis, where the points are distributed randomly in y-direction to make individual points distinguishable.

We analyze the collected target site positions by conducting a kernel density estimation with a box kernel and a bandwidth of 100. In Figure 7, we plot the density estimates against the relative position to the start codon (left) and against the relative position to the TSS (right), where the density estimates for the positive and negative set are plotted as a green and red curve, respectively.

Considering the relative position to the start codon, we find a clear enrichment of positive target sites compared to negative target sites in a region reaching from the start codon approximately 300 bp upstream. At regions farther than 400 bp upstream of the start codon, the density of false positive predictions according to the microarray experiments is consistently greater than the density of the true positives.

We also find a pronounced positional preference relative to the transcription start site as can be observed from the right panel of Figure 7. A substantial fraction of true positive predictions is located in a region extending approximately from 300 bp upstream to 200 bp downstream of the TSS. Again, we find a greater density of negatives than positives at positions farther than 400 bp upstream of the TSS. For many genes, the distance between TSS and start codon is at most 200 bp, i.e., many genes have 5′ untranslated regions of at most 200 bp. Hence, for these genes positions farther than 200 bp downstream of the TSS are not considered in the predictions and we generally find a low number of predicted target sites at distances greater than 200 bp.

We repeat this analysis for rank cutoffs of 100 and 500 with highly similar results (data not shown).

In the following, we investigate whether this strong positional preference may be exploited to reduce the number of false positive predictions. To this end, we use TALgetter to predict target sites in two modified sets of upstream sequences. First, we predict target sites in the 300 bp upstream sequences relative to the start codon of all *O. sativa* genes. Second, we extract for these genes the sequences from 300 bp upstream of the TSS to 200 bp downstream of the TSS or the start codon, whichever comes first. As for the benchmark study of section *Comparison to Target Finder*, we consider rank cutoffs of 10, 20, 50, and 100 on the predictions for a single TAL effector and join the sets of predictions according to the set of TAL effectors expressed by the *Xanthomonas* strain studied in the corresponding microarray experiment.

In supplementary Figure S6, we present the results of this analysis in complete analogy to Figure 1. We find that the restriction of predictions to 300 bp upstream the start codon does not improve the overall prediction performance. We even observe a decrease of prediction performance for 11 of the 32 combinations of data set and rank cutoff, while an improvement can only be found in 10 cases. We find an improvement if we restrict predictions to the [−300,200]-region around the TSS. Here, we observe an improvement of prediction performance for 17 of the 32 combinations, whereas performance decreases only in 5 cases. Hence, we might conclude that true target sites of TAL effectors are preferentially located at most 300 bp upstream and at most 200 bp downstream of the TSS, and that exploiting this positional preference for predictions by TALgetter increases the number of true positive predictions for relevant rank cutoffs. In supplementary Figure S7, we present and discuss models to explain this positional preference. Since we expect that the discovered positional preference is a general characteristic of functional TAL effector target sites, limiting the search region to the [−300,200]-region around the TSS should reduce the number of false-positives of any approach for TAL effector target site prediction.

In Figure 7, we additionally recognize that the peak of true positive predictions is not centered at the TSS but approximately 50 bp upstream. Since this peak is located in close vicinity to the preferred location of core promoter elements like the TATA-box or the TC-box [58], we scrutinize the relationship of TAL effector target sites and core promoter elements in the next section.

### Relationship to core promoter elements

As a first core promoter element, we consider the canonical TATA-box with consensus TATAWA [58], [59]. Approximately 14% of the *O. sativa* genes contain a canonical TATA-box in a preferred distance of 39 to 26 bp upstream of the TSS. Genes containing a canonical TATA-box often belong to the group of highly expressed genes [58], [60].

We find a canonical TATA-box in the promoters of 1445 of the 10903 unique predicted target genes among the top 200 predictions for all TAL effectors considered. This is in well accordance to the rate of 14% reported by Bernard *et al.* [58]. Splitting TATA-related and TATA-less predicted target sites into positives and negatives as described in the previous section, we find 142 TATA-related predictions among the positives (38.4%), whereas 288 positive predictions belong to TATA-less genes. For the set of negative predictions, we find 1303 TATA-related (12.4%) and 9170 TATA-less predictions. Hence, the TATA-related predictions are considerably enriched in the set of positive compared to the negative predictions (odds ratio 3.5). This result is highly significant yielding a p-value below 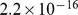 in a one-sided Fisher's exact test, which is the smallest possible p-value due to computational precision.

However, TATA-containing genes might be generally enriched in the set of up-regulated genes regardless of TAL effector target sites. Since genes containing a canonical TATA-box are often highly expressed, this could be an effect of the required log-fold change of 1 for experiments 24 hpi. Indeed, the enrichment of TATA-containing genes among all up-regulated genes is highly significant (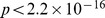) with an odds ratio of 3.6. Hence, the enrichment of TATA-containing genes in the set of positive predictions is not greater than the enrichment of TATA-containing genes in all up-regulated genes.

Nonetheless, there could be a functional relationship between transcriptional activation by a subset of TAL effectors and the presence of a TATA-box. For instance, some TAL effectors might substitute the TATA binding protein and acquire the transcriptional machinery independently. The latter explanation is supported by known TAL effector target sites that overlap a TATA-box including the known target sites of AvrBs3 [2], PthXo3 [49], AvrXa7 [49], and PthXo6 [7]. The TAL effector AvrBs3 shifts the transcription start site of target genes and it has thus been speculated that TAL effectors might functionally mimic the TATA-box binding protein [10], [46], [61]. In contrast, several TAL effectors which recognize adjacent DNA boxes in an artificial target promoter primarily directed gene expression from the same original start site [44]. Therefore, it is likely that additional plant promoter elements contribute to TAL effector-mediated gene induction. In the following, we investigate if the predictions of TALgetter support a direct binding of TAL effectors to the TATA-box.

In the subset of 142 positive target sites in TATA-containing genes, we find 40 target sites (28%) that overlap the putative TATA-box. In contrast, only 134 of the 1303 (10%) TATA-related negative predictions overlap with the TATA-box. This enrichment is significant, yielding a p-value of 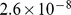 in a one-sided Fisher's exact test. Only 33 of the 142 positive targets sites (23%) and 308 of the 1303 negative target sites (24%) are predictions for one of the 13 TAL effectors having TATAWA in their binding consensus. Hence, the enrichment of target sites overlapping the TATA-box can not be explained by an enrichment of TAL effectors with TATAWA in their binding consensus among the positive predictions.

Interestingly, 26 of the 40 TATA-overlapping target sites directly start with the TATA-box, and 12 overlap the TATA-box with an offset of 2, i.e., start with nucleotides 3 to 6 of the TATAWA consensus. Of the remaining two predicted target sites, one overlaps the TATA-box with an offset of 4, and one contains the TATA-box in the middle of the target site. The large number of TAL effector target sites overlapping TATA-boxes might entail an evolutionary advantage for *Xanthomonas* strains, since mutations in the TATA-box would lead to a change of the transcriptional behavior of the downstream gene and, hence, are disadvantageous for the host plant [49]. In addition, it might help to correctly position the TAL effector with respect to other promoter elements neighboring the TATA-box (cf. supplementary Figure S7).

In addition to the canonical TATA-box, we also examine the enrichment of TATA-variants [58], which are often found in the promoter sequences of housekeeping genes. Interestingly, we do not find an enrichment of genes containing TATA-variants (not including the canonical TATA-box) in the set of positives, where the corresponding p-value of 0.48 in Fisher's exact test is far from significant. Finally, we consider the TC-box [58], i.e., TTCTTC and variants, located in a similar distance to the TSS as the TATA-box. In this case, the enrichment of genes containing a TC-box in their promoters among the positives is not clearly significant (p = 0.031).

We might suspect that the relationship to core promoter elements, especially the observed overlap of predicted target sites with the canonical TATA-box, is the only reason for the positional preference of TAL effector target sites described in the previous section. However, if we remove all genes that contain a canonical TATA-box, a TATA-variant, or a TC-box from the sets of positive and negative genes, and repeat the kernel density estimation for the remaining sets, the overall picture remains unchanged (cf. supplementary Figure S5).

**Figure 8:**
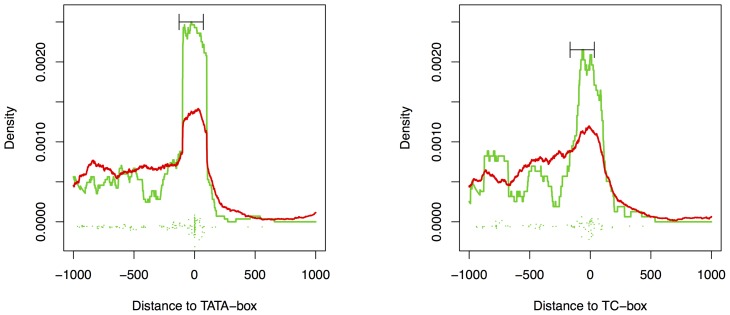
Positional preference of TAL effector target sites relative to the TATA-box (left) and TC-box (right). The estimated density of positions from the positive set is plotted as a green line, while the density of the negatives is plotted in red. The green points at the bottom of the plots represent the distribution of positions from the positive set along the x-axis, where the points are distributed randomly in y-direction to make individual points distinguishable.

In Figure 8, we finally investigate the relative position of target sites to the core promoter element in TATA-box containing (left) and TC-box containing (right) promoters. Considering the set of genes containing a canonical TATA-box, we find a sharp cluster of target sites in close vicinity to and often overlapping the TATA-box, which can be recognized from the individual positions plotted as green points in the lower part of the plots. This again reflects that direct binding to the TATA-box might constitute one potential mode of TAL effector function. The majority of the remaining target sites is located upstream of the TATA-box. For the TC-box containing genes, we observe a broader cluster of target sites around the positions of the TC-box. Similar to the TATA case – and the set of all target sites – we find the remainder of target sites preferentially located upstream of the TSS.

In summary, we find an enrichment of genes with a promoter containing a canonical TATA-box among the predicted TAL effector targets, but a similar enrichment can be found for all up-regulated genes. Within the subset of TATA-containing genes, the number of target sites that overlap the canonical TATA-box is significantly enriched. The most conclusive explanation of this observation is a functional relationship between transcriptional activation by TAL effectors and the TATA-box.

### Predicted target sites with likely biological relevance

In the following, we present and discuss a selection of putative target sites of TAL effectors in rice (*O. sativa* ssp. *japonica*) and sweet orange (*C. sinensis*). For *O. sativa*, we use the refined search region from 300 bp upstream to 200 bp downstream the TSS or the start codon, whichever comes first (cf. *Positional preference of target sites*). To limit false positives, we only study TAL effectors where gene expression data are available. Promising targets show a low rank in the TALgetter predictions and a significantly induced gene expression in microarray studies. Different TAL effectors are known to target the same or related plant genes which indicates that these host genes constitute major virulence targets, and that the pathogen has evolved different TAL effectors to target them [62]. Therefore, we consider it meaningful, if we predict novel targets that are either related to known virulence targets or a common target of different TAL effectors. We list a selection of predicted target sites in Tables 5 and 6, while a complete list is available as supplementary Table S2.

**Table 5:** Predicted targets in *O. sativa*.

No.	TAL effector	Locus	Description	Rank	Support (log-fold change)
**SWEETs**					
1*	PthXo1	Os08g42350	*OS8N3*, nodulin MtN3 family	1	PXO99(9.4); PXO99AME2(9.5); XOO(3.3)
2*	TalC	Os11g31190	*OS11N3*, nodulin MtN3 family	1	BAI3(6.2)
3	Tal7b/Tal8b	Os02g30910	nodulin MtN3 family	91	PXO99(4.3)
**Nutrient supply**					
4	AvrXa27/XOO1134_MAFF	Os06g29790	phosphate transporter 1	21	MAFF311018(2.0); PXO99(2.0); PXOAME1(1.8)
5	Tal6a & XOO2158_MAFF	Os06g29790	phosphate transporter 1	1	PXO99(2.0); MAFF311018(2.0)
6	Tal9d & XOO1132_MAFF	Os10g25310	*OsSPX3*, SPX domain containing	50;48	PXO99(3.0); MAFF311018(5.0)
**Small RNAs**					
7*	XOCORF_0460	Os07g06970	HEN1	1	XOC(1.9)
8	Tal9a & XOO1138_MAFF	Os07g06970	HEN1	1	MAFF311018(5.2); PXO99(5.1); XOO(2.3)
**Signal transmission**					
9	Tal7a/Tal8a	Os08g07760	BRI1-associated receptor kinase	4	PXO99(2.8)
10	XOO1998_MAFF	Os08g07760	BRI1-associated receptor kinase	1	MAFF311018(1.8)
11	XOO2127_MAFF	Os01g50370	MAPKKK protein kinase	1	MAFF311018(4.0)
**Transcriptional regulation/DNA binding**					
12*	PthXo6	Os09g29820	bZIP TF domain containing	2	PXO99(6.9); PXO99AME2(7.1)
13*	PthXo7	Os01g73890	TFIIA gamma chain	2	PXO99(4.6); XOO(1.5)
14	Tal9b	Os06g46366	zinc finger, C3HC4 type	2	PXO99(1.3)
15	XOO1136_MAFF	Os06g09310	zinc finger, C3HC4 type	47	MAFF311018(2.8)
16	Tal2a	Os12g24490	zinc finger, C3HC4 type	28	PXO99(4.0)
17	Tal9e/XOO2001_MAFF	Os04g41229	helix-loop-helix DNA-binding domain	3	PXO99(1.6); MAFF311018(2.4)
18	XOO2865_MAFF	Os07g48450	no apical meristem protein	11	MAFF311018(3.0)
19	Tal5a	Os04g43560	no apical meristem protein	2	PXO99(1.4)
20	XOO1996_MAFF	Os04g52810	no apical meristem protein	6	MAFF311018(3.0)
**Other**					
21	AvrXa10	Os08g09040	Cupin domain containing	41	PXO86(4.5)
22	AvrXa10	Os08g09010	Cupin domain containing	38	PXO86(4.1)
23	Avrpth3	Os06g46500	monocopper oxidase	7	XOC(3.0)
24	Tal4 & XOO2129_MAFF	Os12g24320	ATPase 3	23;16	PXO99(2.7); MAFF311018(3.3)
25	Tal7b/Tal8b	Os01g40290	expressed protein	1	PXO99(3.8); XOO(1.0)
26	Tal9d & XOO1132_MAFF	Os08g05910	peptide transporter PTR2	1	PXO99(2.3); MAFF311018(2.0)

List of predicted targets in *O. sativa*. For each predicted target, we list the name of the TAL effector, the locus ID and the description of the targeted gene, the rank among the predictions of TALgetter for the specific TAL effector, and the list of microarray experiments that support this target. The corresponding log-fold changes observed in the microarray experiments are given in parentheses. TAL effectors with a common target site are listed in the same row. If both TAL effectors have identical RVD sequences, they are separated by a slash. Otherwise, they are separated by an ampersand. Known target sites are marked with an asterisk.

**Table 6:** Predicted targets in *O. sativa* (2).

No.	TAL effector	Distance to start codon	Distance to TSS	Target site sequence
**SWEETs**				
1*	PthXo1	226	79	TGCATCTCCCCCTACTGTACACCAC
2*	TalC	296	91	CATGCATGTCAGCAGCTGGTCAT
3	Tal7b/Tal8b	141	−35	GCTCCTCCTCCTTTCTCCACT
**Nutrient supply**				
4	AvrXa27/XOO1134_MAFF	290	269	TAGCTAGGGGAATCCATG
5	Tal6a & XOO2158_MAFF	332	314	TATAAGTGACAGCCCTCCCCT
6	Tal9d & XOO1132_MAFF	241	118	TAAATTCTCTCCAT
**Small RNAs**				
7*	XOCORF_0460	200	10	TCCCCCTCGCTTCCCTT
8	Tal9a & XOO1138_MAFF	185	−1	TCCCTTCCCTAAACCCCACTT
**Signal transmission**				
9	Tal7a/Tal8a	257	−8	TATAAAGCGAGGCGACGAA
10	XOO1998_MAFF	255	−8	TATAAAGCGAGGCGACGAACT
11	XOO2127_MAFF	142	31	TATATAAACGCACACAAGCGCT
**Transcriptional regulation/DNA binding**				
12*	PthXo6	112	31	TATAAAAGGCCCTCACCAACCCAT
13*	PthXo7	446	30	TATAATCCCCAAATCCCCTCCTC
14	Tal9b	68	−47	TCCAGTTCTCCTCCCCTGAGCTTCTCCC
15	XOO1136_MAFF	115	70	TCCGGCTACTCTCCCCCACGTAGCCGCC
16	Tal2a	82	−56	TATGTGTACAAACATT
17	Tal9e/XOO2001_MAFF	53	−148	CGCAGCGCCCCCGCGCGGAGAAGCT
18	XOO2865_MAFF	355	267	TAGATATAGATAGATAGATAT
19	Tal5a	225	66	TAGCTCGCTTGGCCCCT
20	XOO1996_MAFF	898	46	TATCTAGCTAAATCTCCAT
**Other**				
21	AvrXa10	112	51	TATATAAACACATAAAT
22	AvrXa10	117	31	TATATAAGCACATCAAT
23	Avrpth3	409	271	TACATACTCCACCGCGTA
24	Tal4 & XOO2129_MAFF	192	157	TAGGAAAAATGGTACTC
25	Tal7b/Tal8b	81	32	TATATACCTCGTTTCTCCAGG
26	Tal9d & XOO1132_MAFF	944	56	TAGATTCTCTCCCT

List of predicted targets in *O. sativa*. For each predicted target, we list the name of the TAL effector, the distance from the 3′ end of the target site to the start codon, the distance from the initial position of the target site to the transcription start site (TSS), and the sequence of the target site. Known target sites are marked with an asterisk.

The first group of targets we consider belongs to the family of nodulin *MtN3* genes, of which several members from *A. thaliana* and *O. sativa* encode functional sucrose/glucose transporters (SWEETs) [63], [64]. Some *SWEET* genes have been identified as susceptibility (*S*) genes whose induction is essential for a successful bacterial infection [65]. TAL-mediated *SWEET* gene induction and the resulting elevated export of sugars from plant cells is believed to support bacterial proliferation. In pepper, the *MtN3*-homolog *UPA16* is induced by the TAL effector AvrBs3 [10]. The *O. sativa SWEET* gene *Os8N3* (Os08g42350) is a known target of PthXo1 [65], which is predicted by TALgetter on rank 1 and shows log-fold changes above 1 in three of the microarray experiments. Similarly, the best prediction for the TAL effector TalC is the *SWEET* gene *Os11N3* (Os11g31190), a known target as well [9]. *Os11N3* is also a known target of PthXo3 and AvrXa7 [49], however their target sites are predicted on a much higher rank (cf. section *Recovering known target sites*).

In addition to these known target sites, we find a novel *SWEET* as putative virulence target of Tal7b and Tal8b, which are identical TAL effectors encoded by a duplicated gene in the genome of *Xoo* 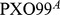 [56]. The predicted target gene of Tal7b/Tal8b is Os02g30910, which yields a log-fold change of 4.3 in the PXO99 experiment. The corresponding target site sequence is shown in the fourth column of Table 5. Notably, the nucleotide at position 0 of this predicted target site is G instead of the usually required T. A few exceptions to the invariable initial T have been reported in natural TAL effector target sites (C for TalC in *Os11N3* [9]; A for AvrBs3 in *UPA25* [10]) and the potential Tal7b/Tal8b target site might be another exception. Like *Os8N3* and *Os11N3*, Os02g30910 belongs to the clade III of the *SWEET* family, which is the only known sub-family that encodes sucrose transporters, indicating that this specific function is important for *Xoo* [64,64]. However, a gene with unknown function (Os01g40290, no. 25) is predicted by TALgetter for Tal7b/Tal8b on rank 1. Since this gene yields a log-fold change above 1 in two of the microarray experiments and has been predicted as a target of Tal7b/Tal8b before [3], it is an alternative target candidate.

The second group of targets also addresses the nutrient supply of the pathogen. We identify several putative TAL effector targets that are related to phosphate metabolism. *Xoo* mutants impaired in the utilization of phytic acid, a storage form of phosphate, are impaired in virulence [66] indicating that the supply of phosphate is important for a successful *Xoo* infection. The phosphate transporter Os06g29790 is predicted as a putative target of three TAL effectors, namely AvrXa27 (RVDs identical to XOO1134_MAFF), Tal6a, and XOO2158_MAFF with the latter two TAL effectors differing by only one RVD. Os06g29790 is predicted by TALgetter on rank 1 for Tal6a and XOO2158_MAFF, and rank 21 for AvrXa27/XOO134_MAFF, and is supported by two and three microarray experiments, respectively. The predicted target sites of Tal6a and AvrXa27 do not overlap, and the common target is thus not due to general similarities between these TAL effectors. AvrXa27 can also trigger resistance due to induced expression of the resistance gene *Xa27*, but only in *O. sativa* ssp. *indica* and not in ssp. *japonica* [67]. Tal9d and XOO1132_MAFF differ in one RVD and are predicted to target the promoter of Os10g25310 which encodes *OsSPX3*, an SPX domain containing negative regulator involved in tolerance to phosphate starvation [68]. High induction of *OsSPX3* after *Xoo* infection is supported by two microarray experiments. An alternative target of Tal9d and XOO1132_MAFF with slightly lower over-expression but rank 1 among the TALgetter predictions is Os08g05910 (no. 26), a peptide transporter.

The third group of target genes contains *HEN1*, a small RNA pathway component that contributes to plant immune responses [69]. *HEN1* methylates the 3′ terminal nucleotide of all classes of small RNA (sRNA) duplexes thereby promoting sRNA stability [70]. The *HEN1* gene is a known target of XOCORF_0460 (Tal1c) and a previously predicted target of Tal9a [3]. In addition, we identified *HEN1* as a target of XOO1138_MAFF, a TAL effector which differs from Tal9a in 5 of 20 RVDs. *HEN1* yields rank 1 among the TALgetter predictions for all three TAL effectors and is up-regulated in 3 different microarray experiments with *Xanthomonas* strains that express either Tal9a or XOO1138_MAFF. This demonstrates that TAL effectors interfere with the sRNA homeostasis of the host cell. This example also demonstrates that *Xoo* and *Xoc* TAL effectors can have common targets and thus common infection strategies despite their different modes of infection as vascular and leaf mesophyll pathogens, respectively.

The fourth group of targets is related to signal transmission. The TAL effectors Tal7a/Tal8a and XOO1998_MAFF have overlapping predicted target sites in the promoter of Os08g07760 with rank 4 and 1, respectively, and two different supporting microarray experiments. This target gene encodes a putative brassinosteroid-insensitive1 associated receptor kinase (BAK1) ortholog from rice. BAK1 is a leucine-rich repeat receptor-like kinase that is involved in both, brassinosteroid and pathogen signal perception [71]. In addition, XOO2127_MAFF is predicted to target and strongly induce Os01g50370, a MAPKKK protein kinase. Elevated expression of *BAK1* or MAP kinase pathway components might interfere with signal transmission and cellular responses.

The fifth and largest group of predicted targets contains genes that are related to transcriptional regulation. AvrBs3 induces the pepper basic helix-loop-helix regulator UPA20 to trigger plant cell enlargement and a hypertrophy phenotype [46] demonstrating that TAL effectors can control complex plant responses via induction of regulatory genes. TALgetter predicts the known targets of PthXo6 and PthXo7, the transcription factor TFX1 and the transcription initiation factor TFIIA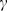 subunit, at rank 2. In addition, three TAL effectors are predicted to target different genes encoding zinc finger proteins (targeted by Tal9b and XOO1136_MAFF differing in 3 RVDs, and Tal2a) or a gene encoding a helix-loop-helix domain containing protein (targeted by Tal9e/XOO2001_MAFF). The predicted targets of XOO2865_MAFF (Os07g48450), Tal5a (Os04g43560), and XOO1996_MAFF (Os04g52810) encode no apical meristem proteins (NAC proteins), a large family of plant transcriptional regulators that are involved in diverse developmental and abiotic/biotic stress response processes, including drought tolerance. TALgetter also predicts NAC gene targets for Tal9d (Os05g34830) and XOO2127_MAFF (Os05g10620) for which we have described alternative targets above. Several NAC encoding genes are induced after pathogen infection and some repress defense-related gene expression, rendering them good candidates for TAL effector virulence targets [72], [73].

The sixth group of predicted targets comprises members with diverse function. AvrXa10 is predicted to induce two target genes (Os08g09040, Os08g09010) and XOO2127_MAFF one target gene (Os08g13440) that encode cupin domain-containing proteins. The cupin superfamily includes functionally diverse proteins that can be involved in transcriptional regulation, seed storage, enzymatic reactions to protect plants from oxidative stresses, and pathogen infection [74]. For AvrPth3 TALgetter predicts a monocopper oxidase (Os06g46500). It has been reported before that *Xanthomonas* influences the defense of rice plants by manipulating copper transport [75]. Tal4 and XOO2129_MAFF, which differ in one RVD, are predicted to induce a gene encoding a putative ATPase with unknown function.

We also use TALgetter to predict TAL effector target sites for *Xanthomonas axonopodis* pv. *citri* (*Xac* 306) in *Citrus sinensis*. A selection of predicted target sites of the four TAL effectors of *Xac* 306, namely PthA1, PthA2, PthA3, and PthA4, is presented in Tables 7 and 8, while the complete list of predicted target sites is available as supplementary Table S3. The predicted target of PthA1 is a late embryogenesis-abundant (LEA) protein (orange1.1g027210m). This family of proteins is often related to drought [76]. However, members of this family have also been reported to be metal-binding [77], which might indicate a role in copper transport [75]. For PthA2, TALgetter predicts a target gene from the Tetratricopeptide repeat (TPR)-like superfamily. Interestingly, it has been reported that PthA2 and PthA3 interact with TPX, which contains a TPR domain as well and is related to protein folding and activation, in *Citrus* [78]. Hence, PthA2 might play a role in supplying such an interactor to PthA2 and PthA3. The predicted target of PthA3 is a RAN GTPase, which might play a role in signal transduction. A LOB domain-containing protein (orange1.1g026556m) is the best target predicted by TALgetter for PthA4. According to gene expression data, this gene is highly up-regulated and achieves a log-fold change of 5.7. For these two reasons, we consider this the most promising candidate of the four *Xac* 306 TAL effectors. LOB domain containing proteins have been shown to act as transcription factors [79].

**Table 7:** Predicted targets in *C. sinensis*.

No.	TAL effector	Locus	Description	Rank	log-fold change
1	PthA1	orange1.1g027210m	LEA hydroxyproline-rich glycoprotein	10	2.2
2	PthA2	orange1.1g015673m	Tetratricopeptide repeat (TPR)-like superfamily	17	1.4
3	PthA3	orange1.1g027607m	RAN GTPase 3	10	1.4
4	PthA4	orange1.1g026556m	LOB domain-containing 1	1	5.7

List of predicted targets in *C. sinensis*. For each predicted target, we list the name of the TAL effector, the locus ID and the description of the targeted gene, the rank among the predictions of TALgetter for the specific TAL effector, and the corresponding log-fold change observed in the microarray experiment.

**Table 8:** Predicted targets in *C. sinensis* (2).

No.	TAL effector	Distance from start codon	Target site sequence
1	PthA1	110	TATATACACACACACCCT
2	PthA2	103	TATACACTTATTTTAAT
3	PthA3	700	TCCATATCTTTAAAACC
4	PthA4	93	TATAAACCCCTTTTGCCTT

List of predicted targets in *C. sinensis*. For each predicted target, we list the name of the TAL effector, the distance from the 3′ end of the target site to the start codon, and the sequence of the target site.

### Predicted target sites can be functional

**Figure 9:**
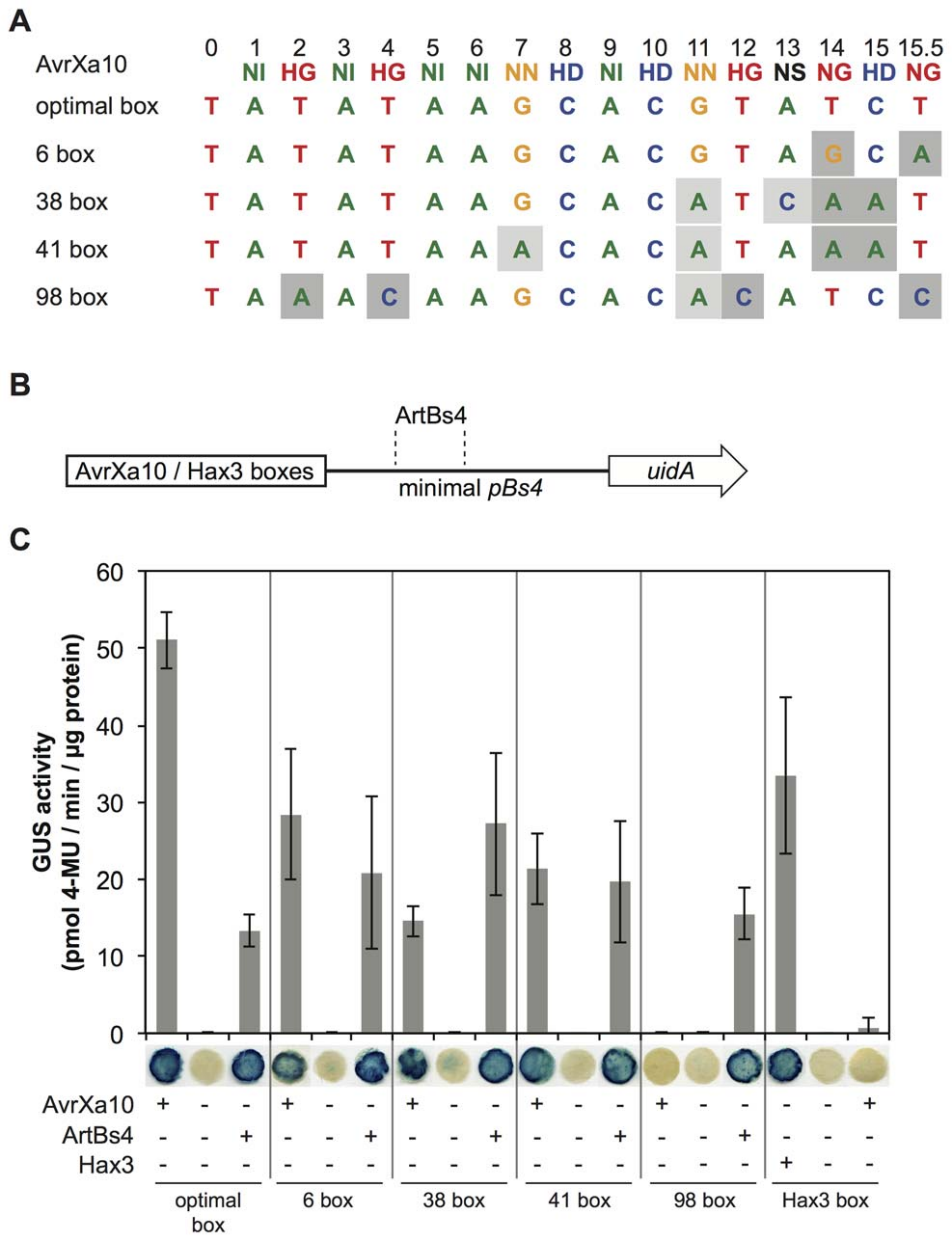
Recognition of predicted target sites by AvrXa10. (A) RVDs of the TAL effector AvrXa10 and predicted target sites. The optimal box is deduced from the known RVD specificites, while box 6, box 38, box 41, and box 98 are TALgetter AvrXa10 target predictions from rice promoters. Mismatches and non-optimal RVD-base pair combinations are shaded in light and dark grey, respectively. (B) AvrXa10 and Hax3 target boxes are cloned upstream of the minimal *pBs4* promoter and a promoterless *uidA* reporter gene. The artificial TAL effector ArtBs4 targets the *pBs4* promoter and is used as control for reporter construct integrity. (C) Specific recognition of target boxes. Reporter constructs are codelivered via *A. tumefaciens* into *N. benthamiana* with (+) and without (−) constructs producing TAL effectors, respectively, and GUS reporter activity was determined two days post inoculation. Error bars indicate standard deviation (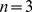 samples). 4-MU, 4-methyl-umbelliferone. Leaf disks are stained with X-Gluc (5-bromo-4-chloro-3-indolyl-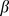-D-glucuronide). A blue color indicates reporter gene activity.

We aim to experimentally test, if the target sites predicted by TALgetter are valid targets for the corresponding TAL effector. For this, we analyzed the TAL effector AvrXa10, for which the target specificity has been experimentally verified [2], but targets have been unknown, so far. Seven putative target rice genes are both, in the top 100 TALgetter predictions and up-regulated in gene expression studies (cf. Tables 5 and 6, supplementary Table S2). Four of these target sites with predictions ranking at position 6, 38, 41, and 98, respectively, are cloned upstream of a minimal promoter and a promoterless reporter gene in a reporter vector and tested for gene activation in a transient reporter assay *in planta* (cf. [2], details in supplementary Text S1), and we present the results of this experiment in Figure 9. Three of the four predicted target sites trigger an AvrXa10-dependent transcriptional activation. Both cupin domain-containing genes which we propose as interesting targets for AvrXa10 (Tables 5 and 6) corresponding to ranks 38 and 41, respectively, therefore contain functional AvrXa10 target boxes. In contrast, the target site with rank 98 has more deviations to the optimal AvrXa10 target site than the other three targets, and this reporter is accordingly not expressed by AvrXa10. Our experimental approach demonstrates that TALgetter can indeed predict functional target sites and that the prediction rank gives an indication for the ability to be recognized by a TAL effector.

### Conclusions

In this paper, we present TALgetter, a new tool for the prediction of TAL effector target sites. TALgetter uses a local mixture model that models binding specificity and importance of RVDs independently. In contrast to previous approaches, the parameters of this model are estimated from training data and, hence, allow for an easy adaptation to new validated target sites.

We demonstrate that TALgetter is able to identify known TAL effector target sites in rice and we show that TALgetter predicts a greater number of TAL effector targets that are consistent with up-regulation after *Xanthomonas* infection than Target Finder in a benchmark study using public and in-house gene expression data. In the benchmark study, a substantial fraction of target sites is uniquely predicted by TALgetter and, hence, these potential virulence targets would have been missed using previous approaches.

Scrutinizing the binding specificities learned by TALgetter, we find that for many RVDs, binding specificities are estimated in accordance to the literature. In addition, we observe gradually decreasing binding specificities for some RVDs, which have also been reported by recent experimental studies. Regarding the concept of RVD importance, we find substantially different parameters for the individual RVDs, which gives indication that different RVDs indeed contribute differently to transcriptional activation by TAL effectors.

In subsequent studies using target sites predicted by TALgetter, we discover a strong positional preference of target sites towards the transcription start site. Most true positive target sites are located within a window from 300 bp upstream to 200 bp downstream the TSS. We demonstrate that exploiting this positional preference for predicting TAL effector target sites further improves the overall prediction performance of TALgetter. This finding is of general value for the computational prediction of TAL effector target sites, since it may also help to reduce the number of false-positive predictions of other approaches.

We also study the relationship of TAL effector target sites to core promoter elements. We show that a considerable number of target sites overlaps with the TATA-box, which indicates that TAL effector binding to the TATA-box – and possibly substituting the TATA binding protein – might constitute one mode of transcriptional activation by TAL effectors. These two findings, positional preference and binding to the TATA-box, reveal new insights into the biology of TAL effector target sites that may aid the understanding of transcriptional activation by TAL effectors. For models to explain this observation, see supplementary Figure S7.

Against this background, we discuss predictions of TALgetter in *Oryza sativa* (rice) and *Citrus sinensis* (sweet orange). Besides several known target sites, TALgetter also predicts promising targets for many TAL effectors with currently unknown targets. We experimentally demonstrate that TALgetter predicts target sites that are functional *in planta*.

We make TALgetter available as a web-application at http://galaxy.informatik.uni-halle.de, which can be used without registration. For confidential analyses, this web-application can also be installed in a local Galaxy server. At http://jstacs.de/index.php/TALgetter, we additionally provide a command line program, which can be easily scripted. Web-application and command line program allow for estimating new model parameters from custom training data to account for the rapid emergence of new TAL effector target sites. Since TALgetter is implemented in the open-source Java library Jstacs, it can be easily extended or modified.

## Supporting Information

Dataset S1 **Training data.** Input sequences in annotated FastA-format used to train TALgetter. The annotation of each DNA sequence contains the RVD sequence of the corresponding TAL effector and the weight of the input sequence used in the training.
(TXT)

Figure S1
**Comparison of TALgetter to Target Finder with a T (Target Finder T), or T or C (Target Finder T/C) at position 0 on public gene expression data.** We consider as performance measure the number of predicted targets that are supported by up-regulation according to gene expression data after *Xanthomonas* infection using a log fold-change of 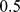. Performance is measured for different rank cutoffs (Top 10, 20, 50, and 100 predictions) on the predictions for each TAL effector.(TIF)

Figure S2 **Summary of the evaluations presented in Figure S1.** For each rank cutoff (10, 20, 50, 100), we count the number of data sets where a prediction program outperforms the other (bars colored identical to program), or both score equally well (bars colored gray).
(TIF)

Figure S3 **Venn diagrams of the predictions of the three programs using a log fold-change of 1 and a rank cutoff of 100.**
(TIF)

Figure S4
**Comparison of TALgetter with binding specificities depending on the individual RVD (dark green) or on amino acid 13 (light green).** Binding specificities and importances of these models are visualized in Figure 5 and 6, respectively. We consider as performance measure the number of predicted targets that are supported by up-regulation according to gene expression data after *Xanthomonas* infection using a log fold-change of 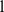. Performance is measured for different rank cutoffs (Top 10, 20, 50, and 100 predictions) on the predictions for each TAL effector.(TIF)

Figure S5 **Positional preference of TAL effector target sites in core promoter element-less upstream sequences relative to the start codon (left) and the transcription start site (TSS, right).** The estimated density of positions from the positive set is plotted as a green line, while the density of the negatives is plotted in red. The whiskers indicate the bandwith of the box kernel used to smooth the curves in a kernel density estimation. The green points at the bottom of the plots represent the distribution of positions from the positive set along the x-axis, where the points are distributed randomly in y-direction to make individual points distinguishable.
(TIF)

Figure S6
**Comparison of TALgetter scanning different types of upstream regions.** i) 1 kb upstream of the start codon, ii) 300 bp upstream of the start codon, and iii) in a region from 300 bp upstream to 200 bp downstream of the transcription start site. We consider as performance measure the number of predicted targets that are supported by up-regulation according to gene expression data after *Xanthomonas* infection using a log fold-change of 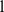. Performance is measured for different rank cutoffs (Top 10, 20, 50, and 100 predictions) on the predictions for each TAL effector.(TIF)

Figure S7 **Models for promoter site preference of TAL effectors.** Natural TAL effector target sites are enriched between −300 and +200 around the natural transcriptional start site. TAL effectors can initiate transcription at TATA box-containing and TATA box-less genes. Often transcriptional initiation starts 40–60 bp following the TAL effector binding site, but the underlying mechanism is unclear. Four models are presented to explain the apparent target site preference of TAL effectors. Combinations of models are possible. (I) The *open chromatin model* reflects that the access of TAL effectors to DNA might be blocked by other proteins. Promoter regions are often less compacted and open areas are typically targeted by transcription factors. (II) The *cooperative model* suggests that TAL effectors execute transcriptional initiation via other factors, some of which might bind to distinct promoter elements. Effective transcriptional initiation thus requires that the TAL effector targets promoter regions that are in an appropriate distance to these promoter elements. (III) The *correct gene product model* emphasizes that natural TAL effectors have likely been selected to upregulate production of functional proteins. This requires that the TAL effector-dependent mRNA allows translational initation at a suitable start codon (e.g. the original). Too early or too late mRNA initiation can lead to use of alternative and potentially out-of-frame ATGs and thereby non-functional products. (IV) The *safe haven model* describes that TAL effectors target DNA regions that are conserved. Some natural TAL effectors have been selected to function as efficient virulence factors which results in selective pressure for the plant to enrich mutations that block TAL effector function. Conserved promoter elements are less likely to change, because mutations also have a deleterious effect on normal gene function. Solid line: DNA; open arrow: open reading frame of a target gene; ATG: original start codon; (ATG): sequences in untranslated regions that can encode for a non-natural start codon if this region is transcribed; +1: natural transcriptional start site.
(TIF)

Table S1 **Tabular overview of training data.** A list of all pairs of RVD sequence and target sequence used for training the TALgetter model. Includes the name of the TAL effector, the name of the target site (if applicable), and GUS activity (if applicable).
(XLS)

Table S2 **Predictions for** ***O. sativa*** **.** List of all target sites predicted by TALgetter for a rank cutoff of 100 that yield a log-fold change greater than 1 in at least one *O. sativa* microarray experiment.
(XLS)

Table S3 **Predictions for** ***C. sinensis*** **.** List of all target sites predicted by TALgetter for a rank cutoff of 100 that yield a log-fold change greater than 1 in the *C. sinensis* microarray experiment.
(XLS)

Text S1 **Supplementary information on priors, parameters, and TAL effectors.** In this file, we explain the prior on the parameters of the statistical model used by TALgetter, define the hyper-parameters of this prior, and list the estimated parameters. In addition, we give the RVD sequences of all TAL effectors used in the studies, and we list the TAL effectors expressed by the *Xanthomonas* strains used in the *O. sativa* experiments.
(PDF)
