# Supplementary figures and images for: Computational Predictions Provide Insights into the Biology of TAL Effector Target Sites
Source: PLoS Comput Biol. 2013 Mar 14;9(3):e1002962. doi: 10.1371/journal.pcbi.1002962 (PMC3597551; doi:10.1371/journal.pcbi.1002962)

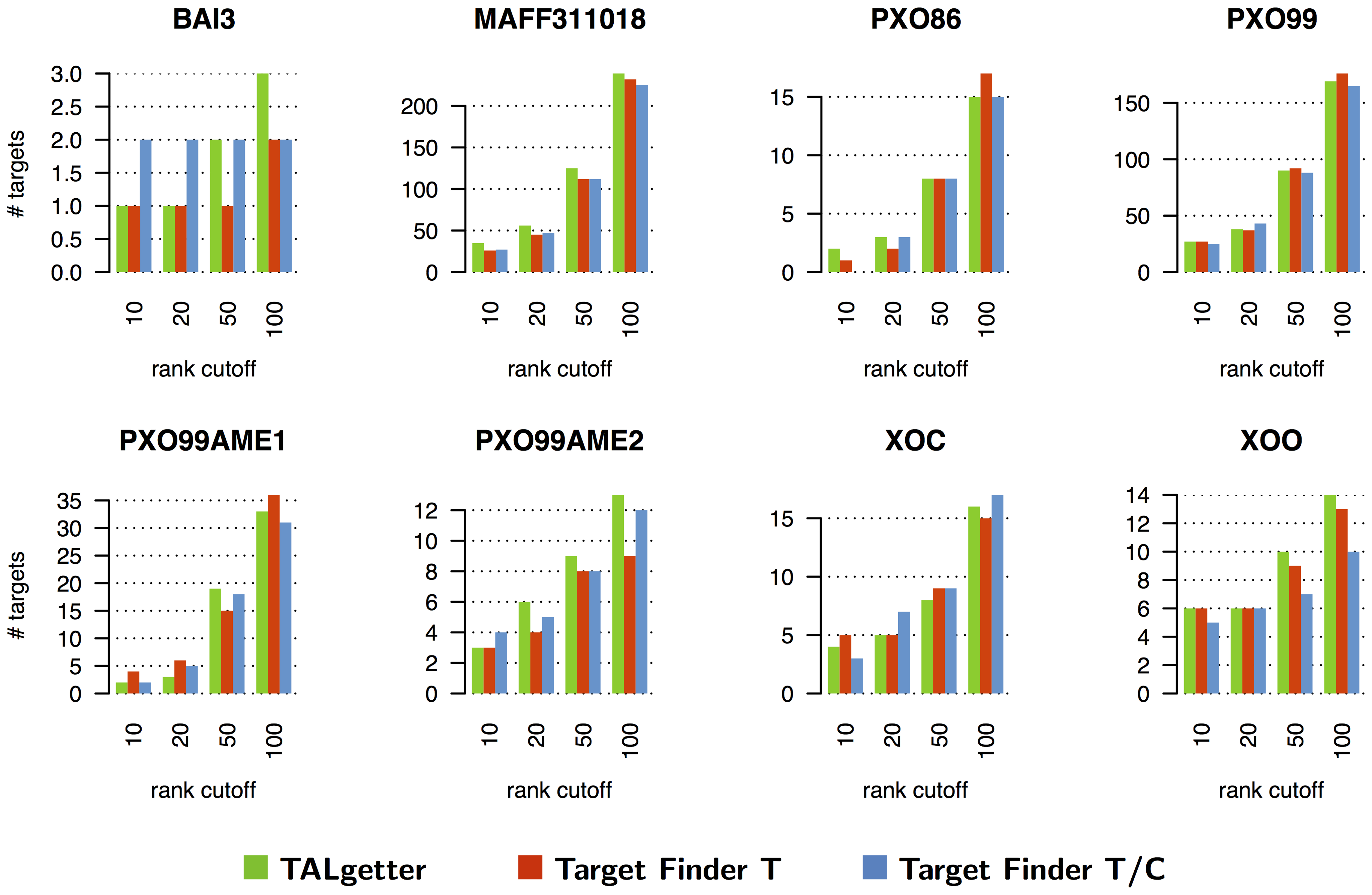

Supplement: Figure S1 — Comparison of TALgetter to Target Finder with a T (Target Finder T), or T or C (Target Finder T/C) at position 0 on public gene expression data. We consider as performance measure the number of predicted targets that are supported by up-regulation according to gene expression data after Xanthomonas infection using a log fold-change of . Performance is measured for different rank cutoffs (Top 10, 20, 50, and 100 predictions) on the predictions for each TAL effector. (TIF) [file pcbi.1002962.s002.tif]

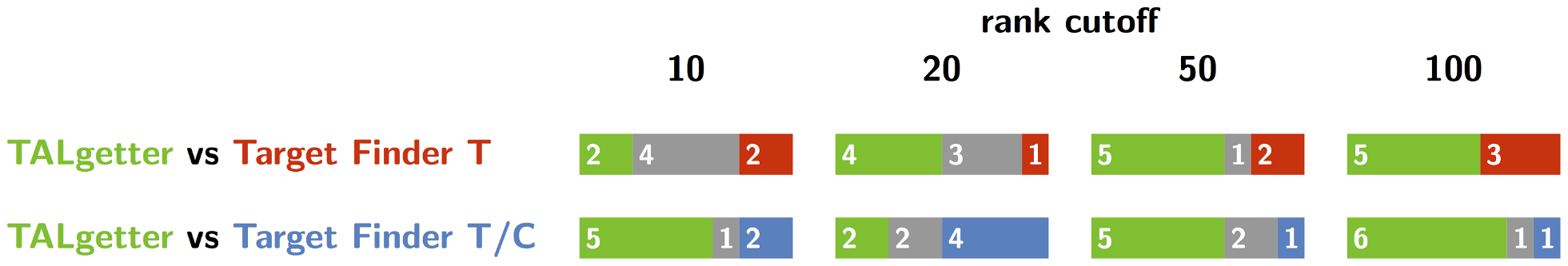

Supplement: Figure S2 — Summary of the evaluations presented in Figure S1. For each rank cutoff (10, 20, 50, 100), we count the number of data sets where a prediction program outperforms the other (bars colored identical to program), or both score equally well (bars colored gray). (TIF) [file pcbi.1002962.s003.tif]

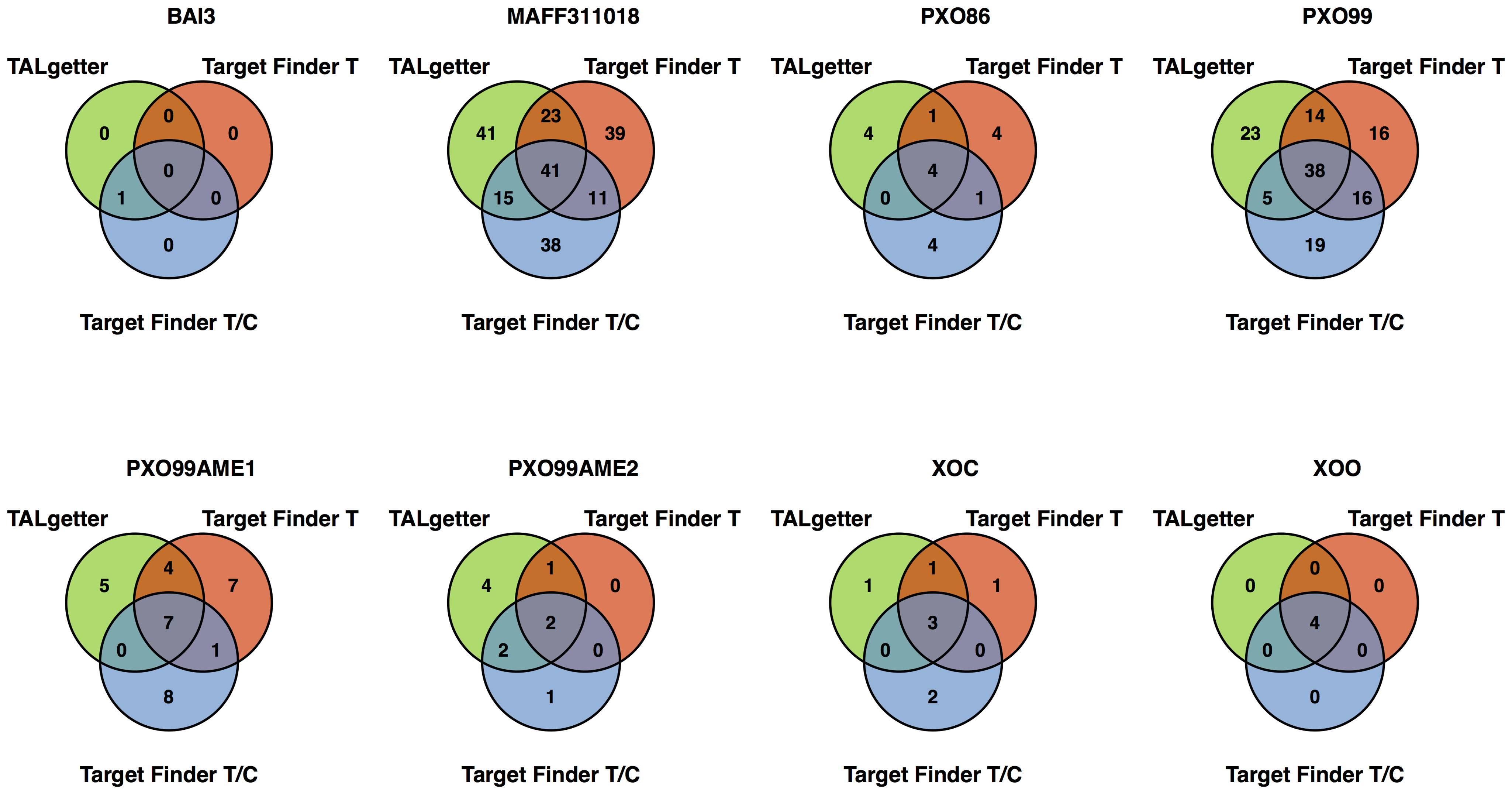

Supplement: Figure S3 — Venn diagrams of the predictions of the three programs using a log fold-change of 1 and a rank cutoff of 100. (TIF) [file pcbi.1002962.s004.tif]

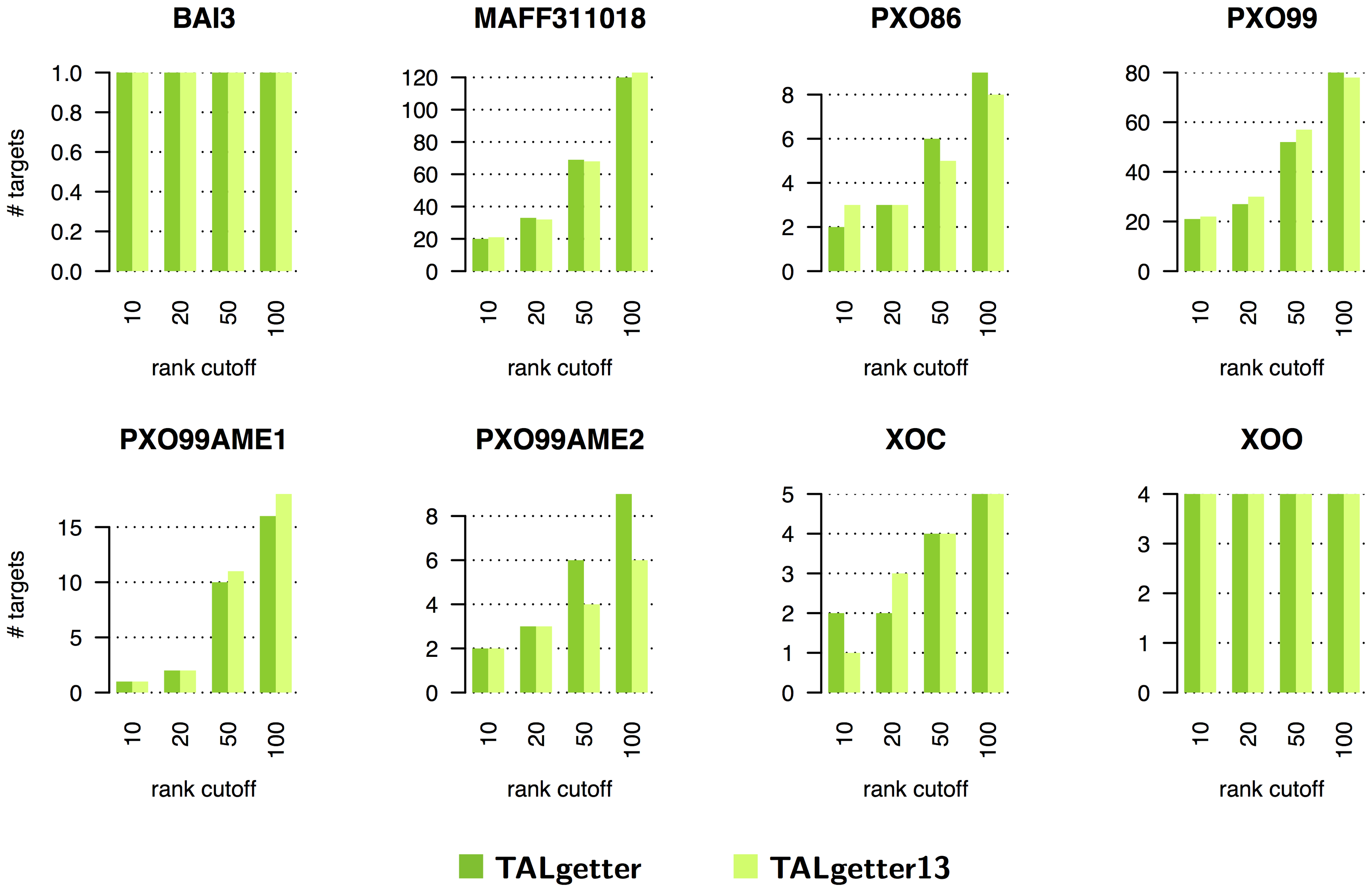

Supplement: Figure S4 — Comparison of TALgetter with binding specificities depending on the individual RVD (dark green) or on amino acid 13 (light green). Binding specificities and importances of these models are visualized in Figure 5 and 6, respectively. We consider as performance measure the number of predicted targets that are supported by up-regulation according to gene expression data after Xanthomonas infection using a log fold-change of . Performance is measured for different rank cutoffs (Top 10, 20, 50, and 100 predictions) on the predictions for each TAL effector. (TIF) [file pcbi.1002962.s005.tif]

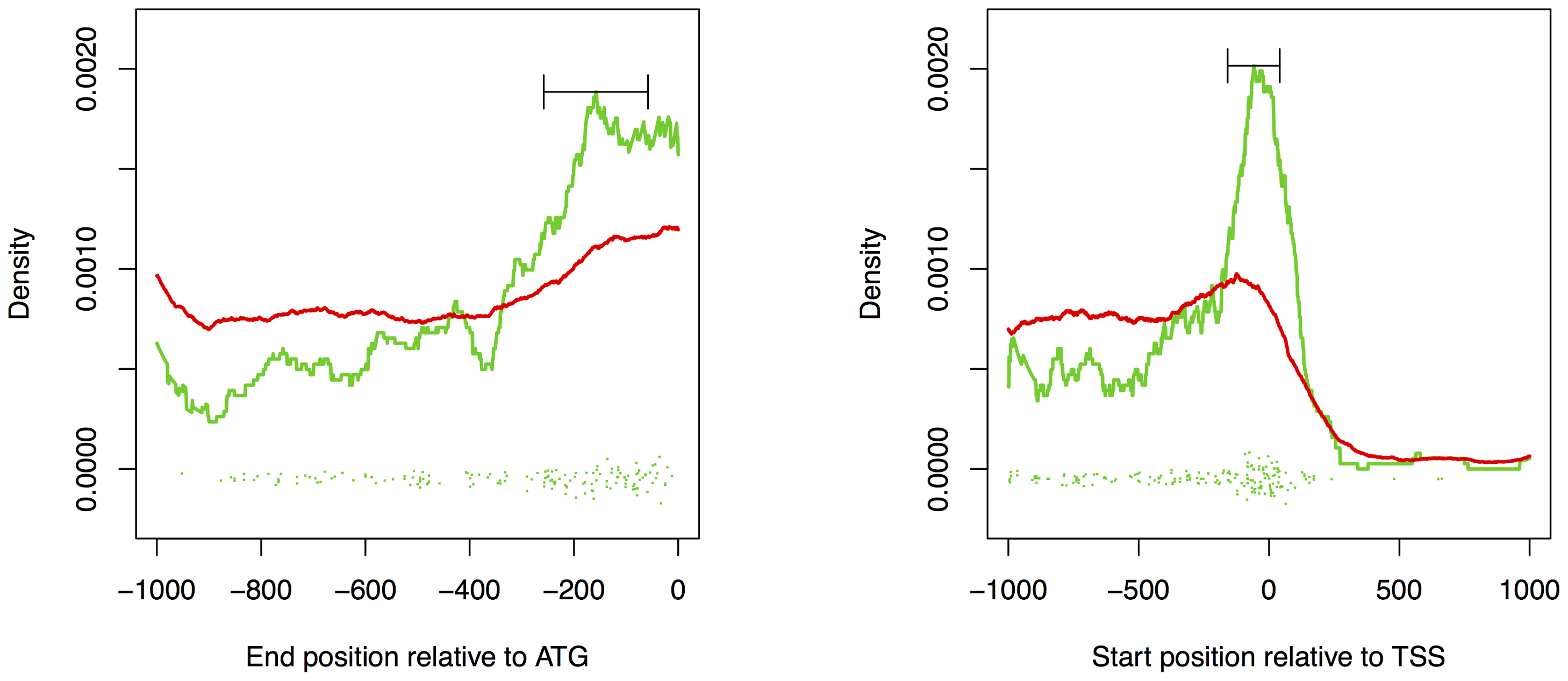

Supplement: Figure S5 — Positional preference of TAL effector target sites in core promoter element-less upstream sequences relative to the start codon (left) and the transcription start site (TSS, right). The estimated density of positions from the positive set is plotted as a green line, while the density of the negatives is plotted in red. The whiskers indicate the bandwith of the box kernel used to smooth the curves in a kernel density estimation. The green points at the bottom of the plots represent the distribution of positions from the positive set along the x-axis, where the points are distributed randomly in y-direction to make individual points distinguishable. (TIF) [file pcbi.1002962.s006.tif]

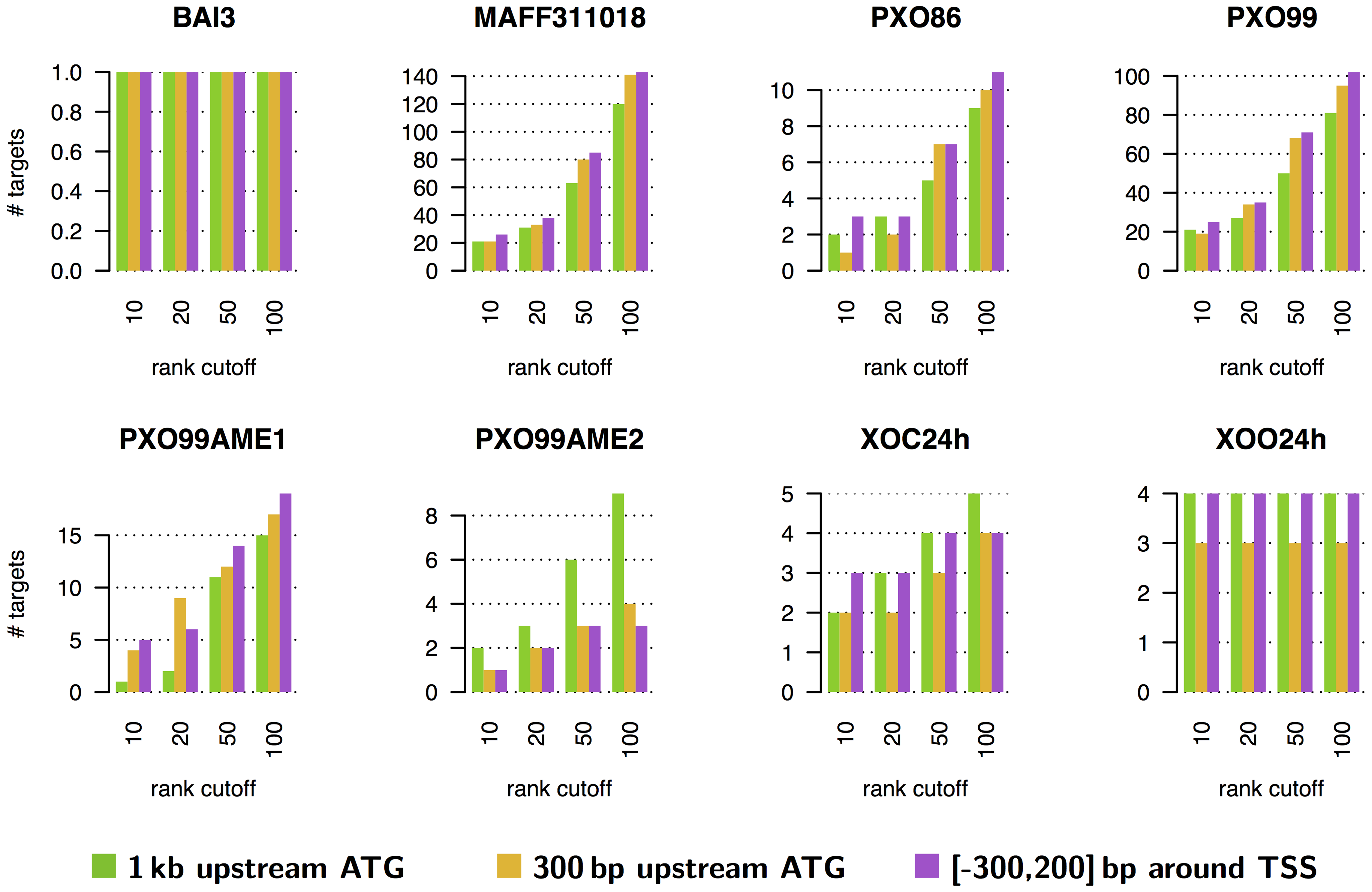

Supplement: Figure S6 — Comparison of TALgetter scanning different types of upstream regions. i) 1 kb upstream of the start codon, ii) 300 bp upstream of the start codon, and iii) in a region from 300 bp upstream to 200 bp downstream of the transcription start site. We consider as performance measure the number of predicted targets that are supported by up-regulation according to gene expression data after Xanthomonas infection using a log fold-change of . Performance is measured for different rank cutoffs (Top 10, 20, 50, and 100 predictions) on the predictions for each TAL effector. (TIF) [file pcbi.1002962.s007.tif]

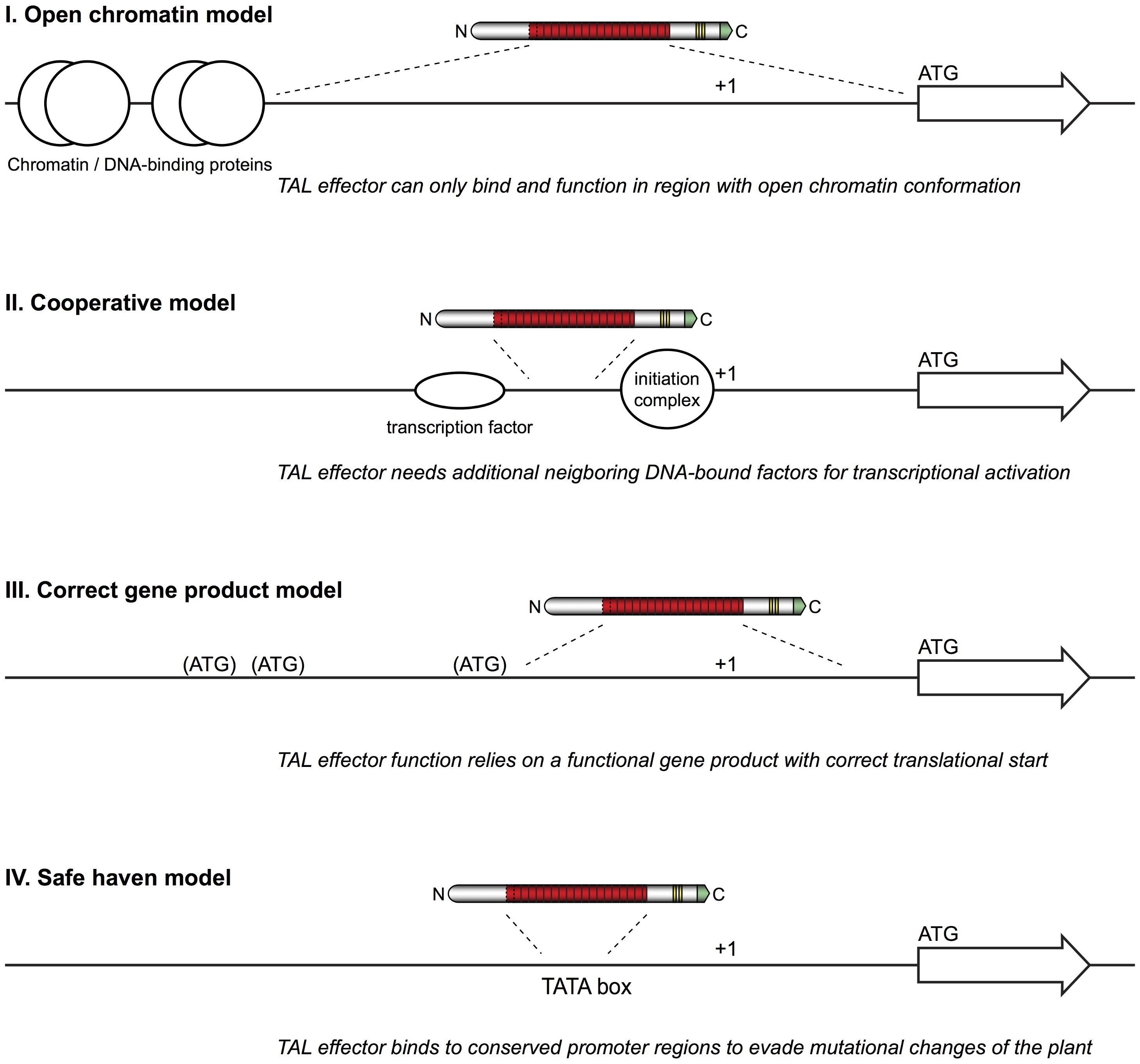

Supplement: Figure S7 — Models for promoter site preference of TAL effectors. Natural TAL effector target sites are enriched between −300 and +200 around the natural transcriptional start site. TAL effectors can initiate transcription at TATA box-containing and TATA box-less genes. Often transcriptional initiation starts 40–60 bp following the TAL effector binding site, but the underlying mechanism is unclear. Four models are presented to explain the apparent target site preference of TAL effectors. Combinations of models are possible. (I) The open chromatin model reflects that the access of TAL effectors to DNA might be blocked by other proteins. Promoter regions are often less compacted and open areas are typically targeted by transcription factors. (II) The cooperative model suggests that TAL effectors execute transcriptional initiation via other factors, some of which might bind to distinct promoter elements. Effective transcriptional initiation thus requires that the TAL effector targets promoter regions that are in an appropriate distance to these promoter elements. (III) The correct gene product model emphasizes that natural TAL effectors have likely been selected to upregulate production of functional proteins. This requires that the TAL effector-dependent mRNA allows translational initation at a suitable start codon (e.g. the original). Too early or too late mRNA initiation can lead to use of alternative and potentially out-of-frame ATGs and thereby non-functional products. (IV) The safe haven model describes that TAL effectors target DNA regions that are conserved. Some natural TAL effectors have been selected to function as efficient virulence factors which results in selective pressure for the plant to enrich mutations that block TAL effector function. Conserved promoter elements are less likely to change, because mutations also have a deleterious effect on normal gene function. Solid line: DNA; open arrow: open reading frame of a target gene; ATG: original start codon; (ATG): [file pcbi.1002962.s008.tif]
