# Supplementary material for: Computational Predictions Provide Insights into the Biology of TAL Effector Target Sites
Source: PLoS Comput Biol. 2013 Mar 14;9(3):e1002962. doi: 10.1371/journal.pcbi.1002962 (PMC3597551; doi:10.1371/journal.pcbi.1002962)
Supplement: Text S1 — Supplementary information on priors, parameters, and TAL effectors. In this file, we explain the prior on the parameters of the statistical model used by TALgetter, define the hyper-parameters of this prior, and list the estimated parameters. In addition, we give the RVD sequences of all TAL effectors used in the studies, and we list the TAL effectors expressed by the Xanthomonas strains used in the O. sativa experiments. (PDF) [file pcbi.1002962.s012.pdf]

Supplementary Text S1

Priors and hyper-parameters

We denote as $Di(\boldsymbol{\theta}|\boldsymbol{\alpha})$ the Dirichlet prior on parameters $\boldsymbol{\theta}$ given hyper-parameters $\boldsymbol{\alpha}$. The complete product-Dirichlet prior is defined as

$$\begin{aligned} Di(\boldsymbol{\theta}|\boldsymbol{\alpha}) \quad &= Di(\boldsymbol{\theta}_0|\boldsymbol{\alpha}_0)\left[\prod_{y\in\mathcal{R}} Di(\boldsymbol{\theta}_{M,y}|\boldsymbol{\alpha}_{M,y})Di(\boldsymbol{\theta}_{D,y}|\boldsymbol{\alpha}_{D,y})\right] \\ &\quad Di(\boldsymbol{\theta}_{I,0}|\boldsymbol{\alpha}_{I,0})\prod_{x\in\mathcal{A}} Di(\boldsymbol{\theta}_{I,1,x}|\boldsymbol{\alpha}_{I,1,x})\prod_{\boldsymbol{c}\in\mathcal{A}^2} Di(\boldsymbol{\theta}_{I,\boldsymbol{c}}|\boldsymbol{\alpha}_{I,\boldsymbol{c}}), \end{aligned}$$

where $\boldsymbol{\theta}_{M,y}$ denotes the parameters of P_M given RVD y, $\boldsymbol{\theta}_{D,y}$ denotes the parameters of P_D given RVD y, $\boldsymbol{\theta}_{I,0}$ denotes the parameters of P_I without context, $\boldsymbol{\theta}_{I,1,x}$ denotes the parameters of P_I with a context x of length 1, $\boldsymbol{\theta}_{I,\boldsymbol{c}}$ denotes the parameters of P_I for the full context $\boldsymbol{c}$ of length 2. The corresponding hyper-parameters are denoted accordingly.

In the implementation, we use natural parameters as defined in [1]. For these parameters, we use a product-Dirichlet prior that is transformed to the natural parameterization as presented in [2].

We define the hyper-parameters according to the consistency conditions necessary for the prior being Bayesian-Dirichlet likelihood equivalent [3, 4] using an equivalent sample size of 16.

$Di(\boldsymbol{\theta}_0|\boldsymbol{\alpha}_0)$

A	C	G	T
1.6	3.2	1.6	9.6

$Di(\boldsymbol{\theta}_{M,y}|\boldsymbol{\alpha}_{M,y})$

y	$\alpha_{M,y,D}$	$\alpha_{M,y,I}$
NI	2.954	6.892
NG	2.954	6.892
NN	8.862	0.985
NS	6.892	2.954
N*	2.954	6.892
ND	4.923	4.923
NK	2.954	6.892
NC	4.923	4.923
NV	4.923	4.923
NA	4.923	4.923
NH	6.892	2.954
HD	8.862	0.985
HG	4.923	4.923
HA	4.923	4.923
H*	4.923	4.923
HH	4.923	4.923
HI	4.923	4.923
HN	6.892	2.954
S*	4.923	4.923
SN	2.954	6.892
SS	4.923	4.923
IG	4.923	4.923
YG	4.923	4.923
NP	6.892	2.954
NT	6.892	2.954
IS	2.954	6.892

$Di(\boldsymbol{\theta}_{D,y}|\boldsymbol{\alpha}_{D,y})$

y	A	C	G	T
NI	2.068	0.295	0.295	0.295
NG	0.295	0.295	0.295	2.068
NN	2.658	0.886	4.431	0.886
NS	2.412	1.378	2.412	0.689
N*	0.295	0.295	0.295	2.068
ND	0.492	3.446	0.492	0.492
NK	0.295	0.295	2.068	0.295
NC	1.231	1.231	1.231	1.231
NV	1.231	1.231	1.231	1.231
NA	0.492	0.492	3.446	0.492
NH	0.689	0.689	4.825	0.689
HD	0.886	6.203	0.886	0.886
HG	0.492	0.492	0.492	3.446
HA	0.985	2.462	0.985	0.492
H*	0.492	0.492	0.492	3.446
HH	1.231	1.231	1.231	1.231
HI	0.492	3.446	0.492	0.492
HN	2.068	0.689	3.446	0.689
S*	1.231	1.231	1.231	1.231
SN	0.295	0.295	2.068	0.295
SS	1.231	1.231	1.231	1.231
IG	0.492	0.492	0.492	3.446
YG	1.231	1.231	1.231	1.231
NP	1.378	1.378	0.689	3.446
NT	2.068	0.689	3.446	0.689
IS	0.738	0.738	0.738	0.738

$Di(\boldsymbol{\theta}_{I,0}|\boldsymbol{\alpha}_{I,0})$

A	C	G	T
1.908	1.908	1.908	1.908

$Di(\boldsymbol{\theta}_{I,1,x}|\boldsymbol{\alpha}_{I,1,x})$

x	A	C	G	T
A	0.477	0.477	0.477	0.477
C	0.477	0.477	0.477	0.477
G	0.477	0.477	0.477	0.477
T	0.477	0.477	0.477	0.477

$Di(\boldsymbol{\theta}_{I,\boldsymbol{c}}|\boldsymbol{\alpha}_{I,\boldsymbol{c}})$

$\boldsymbol{c}$	A	C	G	T
AA	1.669	1.669	1.669	1.669
AC	1.669	1.669	1.669	1.669
AG	1.669	1.669	1.669	1.669
AT	1.669	1.669	1.669	1.669
CA	1.669	1.669	1.669	1.669
CC	1.669	1.669	1.669	1.669
CG	1.669	1.669	1.669	1.669
CT	1.669	1.669	1.669	1.669
GA	1.669	1.669	1.669	1.669
GC	1.669	1.669	1.669	1.669
GG	1.669	1.669	1.669	1.669
GT	1.669	1.669	1.669	1.669
TA	1.669	1.669	1.669	1.669
TC	1.669	1.669	1.669	1.669
TG	1.669	1.669	1.669	1.669
TT	1.669	1.669	1.669	1.669

Estimated parameters of TALgetter

$P(x_0|\boldsymbol{\theta}_0)$

A	C	G	T
0.049	0.100	0.022	0.829

$P(U_\ell = D | y_\ell, \boldsymbol{\theta}_M)$

y_ℓ	$P(U_\ell = D \mid y_\ell, \boldsymbol{\theta}_M)$
NI	0.850
NG	0.820
NN	0.934
NS	0.832
N*	0.615
ND	0.500
NK	0.587
NC	0.500
NV	0.500
NA	0.500
NH	0.837
HD	0.977
HG	0.703
HA	0.500
H*	0.544
HH	0.500
HI	0.500
HN	0.766
S*	0.500
SN	0.363
SS	0.500
IG	0.547
YG	0.500
NP	0.716
NT	0.752
IS	0.300

$P_D(x_\ell | y_\ell, \boldsymbol{\theta}_D)$

y_ℓ	A	C	G	T
NI	0.904	0.087	0.005	0.003
NG	0.005	0.065	0.003	0.926
NN	0.171	0.124	0.675	0.030
NS	0.662	0.210	0.103	0.025
N*	0.015	0.693	0.019	0.272
ND	0.100	0.700	0.100	0.100
NK	0.026	0.024	0.926	0.025
NC	0.250	0.250	0.250	0.250
NV	0.250	0.250	0.250	0.250
NA	0.100	0.100	0.700	0.100
NH	0.038	0.039	0.885	0.038
HD	0.084	0.884	0.006	0.026
HG	0.027	0.128	0.027	0.819
HA	0.200	0.500	0.200	0.100
H*	0.076	0.076	0.076	0.771
HH	0.250	0.250	0.250	0.250
HI	0.100	0.700	0.100	0.100
HN	0.210	0.058	0.675	0.057
S*	0.250	0.250	0.250	0.250
SN	0.069	0.067	0.805	0.059
SS	0.250	0.250	0.250	0.250
IG	0.076	0.076	0.076	0.773
YG	0.250	0.250	0.250	0.250
NP	0.161	0.177	0.062	0.600
NT	0.257	0.059	0.625	0.059
IS	0.250	0.250	0.250	0.250

$P_I(x_0 | \boldsymbol{\theta}_I)$

After the optimization, the estimated parameters are replaced with the parameters of the stationary distribution of $P_I(x_\ell | x_{\ell-2}, x_{\ell-1}, \boldsymbol{\theta}_I)$*, which are listed in the following.*

A	C	G	T
0.291	0.211	0.201	0.297

$P_I(x_1 | x_0, \boldsymbol{\theta}_I)$

After the optimization, the estimated parameters are replaced with the parameters of the conditional stationary distribution of $P_I(x_\ell | x_{\ell-2}, x_{\ell-1}, \boldsymbol{\theta}_I)$*, which are listed in the following.*

x_0	A	C	G	T
A	0.343	0.179	0.193	0.285
C	0.293	0.239	0.186	0.283
G	0.293	0.225	0.226	0.256
T	0.238	0.213	0.203	0.346

$P_I(x_\ell | x_{\ell-2}, x_{\ell-1}, \boldsymbol{\theta}_I)$

$x_{\ell-2}x_{\ell-1}$	A	C	G	T
AA	0.393	0.167	0.176	0.264
AC	0.329	0.229	0.172	0.270
AG	0.330	0.212	0.209	0.249
AT	0.278	0.200	0.196	0.325
CA	0.327	0.218	0.175	0.280
CC	0.299	0.236	0.206	0.258
CG	0.266	0.254	0.252	0.228
CT	0.225	0.276	0.185	0.314
GA	0.311	0.170	0.247	0.272
GC	0.268	0.259	0.207	0.265
GG	0.289	0.245	0.222	0.244
GT	0.231	0.197	0.243	0.329
TA	0.313	0.168	0.188	0.332
TC	0.275	0.235	0.165	0.325
TG	0.279	0.203	0.228	0.290
TT	0.218	0.193	0.200	0.389

RVD sequences of TAL effectors

In the following, we list the RVD sequences of the TAL effectors considered in this paper. The amino acids of the RVD are given in one-letter code and RVDs are separated by dashes. The symbol * indicates a missing amino acid in the repeat.

Avrpth3
NS-HD-NI-NG-NI-HG-NG-HD-NN-NI-NG-HD-NN-NS-NN-NG-NN
Avrpth14
NS-HD-NI-NG-NI-HG-NI-NI-NG-NG-HD-NN-NI-NG-HD-NN-NS-NN-NG-NN
XOCORF_0460
HD-HD-HD-HD-HD-NG-HD-NN-HD-NG-HG-NN-HD-N*-NG-NG
XOCORF_0463
NI-NN-NI-NI-NG-HD-NG-NN-NI-NN-NI-NN-HD
XOCORF_0473
NN-HD-NI-NK-HD-HD-HD-NG-NI-NN-HD-HD-NG
XOCORF_1565
NN-NN-ND-N*-NS-NS-YG-NI-SN-HD-HD-NG-NS-NN-HD-NG
XOCORF_1566
HD-HD-NG-NI-HG-HD-HG-NI-HD-NN-HD-HG-HG-HG-NG-NI-NG
XOCORF_1567
NI-NN-NI-NI-NI-N*-NG-NN-NN-NI-NS-NG
XOCORF_1570
NI-HG-N*-NN-N*-HD-HD-NN-HD-NK-HG-NN-NN-NG-HD
XOCORF_1572
HD-HD-NN-NG-N*-HD-NI-NG-HD-NG-NS-HD-HA-ND-N*-NN-HD-NN-HD-N*-NN-NG-HD
XOCORF_1583
HD-HD-NC-HD-NC-NG-HH-NI-NG-N*-NS-N*-HD-HD-NS-NI-HG-NG
XOCORF_1704

NN-HD-NI-NI-NG-NN-NN-HD-NN-NI-NI-NN-NN-NN-NG-NI-HD-NG
XOCORF_1709
NI-N*-NI-NI-NN-N*-NI-NK-HD-HD-NG-NI-NN-NN-HD-NG
XOCORF_1716
NN-HD-NI-HD-NN-NG-NG-NN-HD-N*-NN-HD-HD-NG
XOCORF_2007
NS-HG-NI-NG-NS-NN-ND-N*-NG-N*-HN-HD-NS-NI-NN-HD-NG-HD-NG
XOCORF_2009
NS-HG-NI-NG-NS-NN-NG-NN-NG-HD-NN-HD-HD-NG
XOCORF_2010
NI-NG-NI-NG-NN-NG-NN-NG-HD-NN-NN-HG-HD-NN-NS-NN-HD-HD-NG-NA-NN-HD-HD-HD-HD-N*
XOCORF_2480
NN-HD-NS-NG-HD-NN-HD-NI-HD-NN-HD-NN-HD-NN-HD-NN-NN-NN-NN-NN-NN-NN-HD-NG
XOCORF_2485
HH-NN-HD-HD-NG-N*-HD-NN-HD-ND-N*-NG-NS-NS-HD-HD-NG
XOCORF_2857
NN-HD-NI-NI-NN-HA-NS-NI-HD-HA-HA-HD-HD-HA-N*
XOCORF_2892
NN-HD-HD-HH-NN-NG-NS-NG-HD-NI-NG-HD-HH-NG-NG-NG-HA-NG
XOCORF_4025
NN-NG-HD-NG-HD-HD-NI-NH-HG-NI-NN-NN-HD-NS-NG-NN-HD-NG-NG-N*
XOCORF_4248
NI-NN-HN-NN-NI-NG-HD-NN-HD-HG-HD-HG-HG-HD-HD-NG
XOCORF_4250
NN-NS-NG-NI-HD-HD-NG-NN-HD-NI-NG-N*-N*-NS-NI-NN-NG-NG
XOCORF_4384
HD-HG-N*-NN-NG-HD-NN-HD-NG-NN-HD-N*-NG-NG
XOCORF_4392
HD-HD-HD-HD-HD-NG-HD-NN-HD-ND-NG-NG-NN-HG-ND-ND-HD-NG
XOCORF_4399
HD-NN-HD-N*-NS-NN-HG-NI-NI-NG-NN-NG-NG
XOCORF_4531
NS-HD-NI-N*-NI-HG-NI-N*-NG-HD-NN-NI-NG-NN-NS-NG-NN
AvrXa27
NI-NN-N*-NG-NS-NN-NN-NN-NI-NN-NI-N*-HD-HD-NI-NG-NG
PthXo1
NN-HD-NI-HG-HD-NG-N*-HD-HD-NI-NG-NG-NI-HD-NG-NN-NG-NI-NI-NI-NI-N*-NS-N*
PthXo6
NI-H*-NI-NN-NN-NN-NN-NN-HD-NI-HD-HG-HD-NI-N*-NS-NI-NI-HG-HD-NS-NS-NG
PthXo7
NI-NG-NI-NI-N*-NN-HD-HD-N*-NI-NI-NI-NG-HD-HG-NN-NS-NN-HD-HD-NG-N*
Tal2a
NI-NG-NN-NG-NK-NG-NI-NN-NI-NN-NI-HD-N*-NS-NG
Tal4
NI-NN-NN-NI-NI-NI-HD-NS-HG-NN-NN-NN-NI-NI-HG-HD
Tal5a
NI-NS-HD-HG-NS-NN-HD-H*-NG-NN-NN-HD-HD-NG-HD-NG
Tal6a
NI-N*-NI-NS-NN-NG-NN-NS-N*-NS-NN-NS-N*-NI-HG-HD-NI-HD-HD-NG

Tal7a
NI-HG-NI-NI-NI-NN-HD-NS-NN-NS-NN-HD-NN-NI-HD-NN-NS-NG
Tal7b
NI-HG-NS-HG-HG-HD-NS-NG-HD-NN-NG-HG-NG-HD-HG-HD-HD-NI-NN-NG
Tal8a
NI-HG-NI-NI-NI-NN-HD-NS-NN-NS-NN-HD-NN-NI-HD-NN-NS-NG
Tal8b
NI-HG-NS-HG-HG-HD-NS-NG-HD-NN-NG-HG-NG-HD-HG-HD-HD-NI-NN-NG
Tal9a
HD-HD-HD-NG-N*-NN-HD-HD-N*-NI-NI-NN-HD-HI-ND-HD-NI-HD-NG-NG
Tal9b
HD-HD-NN-NN-NG-NG-HD-NS-HG-HD-NG-N*-HD-HD-HD-N*-NN-NI-NN-HD-HI-ND-HD-HG-NN-HG-NG
Tal9d
NI-NN-NI-HG-HG-NN-HG-HD-HG-HD-HD-HD-NG
Tal9e
NN-HD-NS-NG-HD-NN-N*-NI-HD-NS-HD-NN-HD-NN-HD-NN-NN-NN-NN-NN-NN-NN-HD-NG
Talc
NS-NG-NS-HD-NI-NG-NN-NG-HD-NI-NN-N*-NI-NN-HD-NG-NI-NN-N*-HD-NN-NG
AvrXa7
NI-HG-NI-NI-NS-HD-NN-HD-HD-HD-NS-N*-N*-HD-HD-NS-NS-NN-NN-NI-NG-NN-NI-N*-NS-N*
AvrXa10
NI-HG-NI-HG-NI-NI-NN-HD-NI-HD-NN-HG-NS-NG-HD-NG
XOO2868_MAFF
NI-HG-NI-NI-NS-HD-NN-HD-HD-HD-NS-N*-N*-HD-HD-NS-NS-NN-NN-NI-NG-NN-NI-N*-NS-NG
XOO1132_MAFF
NI-NN-NI-HG-HG-NV-HG-HD-HG-HD-HD-HD-NG
XOO1136_MAFF
HD-HD-NN-NN-NS-NG-HD-S*-HG-HD-NG-N*-HD-HD-HD-N*-NN-NI-NN-HD-HI-ND-HD-HG-NN-HG-N*
XOO1138_MAFF
HD-HD-HD-NG-N*-NG-HD-S*-HG-NI-NI-NN-HD-NN-ND-HD-NI-HD-HG-NG
XOO1996_MAFF
NI-NS-HD-NG-NS-NN-HD-N*-NN-NN-NI-NG-HD-NG-HD-HD-HD-NG
XOO1998_MAFF
NI-HG-NI-NI-NI-NN-HD-NS-NN-NS-NN-HD-NN-NI-HD-NN-NI-NG-HD-NG
XOO2127_MAFF
NI-N*-NI-HG-NI-NI-NS-HD-NN-HD-NS-NG-SS-HD-NI-NI-NN-NI-NN-NS-NG
XOO2129_MAFF
NI-NN-NN-NI-NI-NI-HD-NS-HG-NN-NN-NN-NI-NI-NG-HD
XOO2158_MAFF
NI-N*-NI-NS-NN-NG-NN-NS-N*-NS-NN-NS-N*-HD-HG-HD-NI-HD-HD-NG
XOO2160_MAFF
NI-HG-NI-NG-HG-HD-NS-NG-HD-NN-NG-HG-NG-HD-HG-HD-HD-NI-NS-HG-HD-NI-N*-NS-NI-NI-HD-HD-N*-NS-N*
XOO2864_MAFF
HD-HD-HD-NS-N*-NN-NS-N*-NS-NN-NI-NN-NI-HG-NI-NI-NS-HD-NN-HD-NS-NS-NN-HD-NN-NN-NN-NN-NN-HD-HD-NG
XOO2865_MAFF

NI-NG-NN-NG-NK-NG-NI-NN-NI-NN-NI-NN-NS-NG-NS-NN-NI-N*-NS-NG
XOO2866_MAFF
NI-HG-NI-NI-HG-HD-NN-HD-HD-HD-NI-NI-NN-NI-HD-HD-HD-HG-NN-NN-HD-NS-NN-HD-NG-NS-N*
XOO1134_MAFF
NI-NN-N*-NG-NS-NN-NN-NN-NI-NN-NI-N*-HD-HD-NI-NG-NG
XOO2001_MAFF
NN-HD-NS-NG-HD-NN-N*-NI-HD-NS-HD-NN-HD-NN-HD-NN-NN-NN-NN-NN-NN-NN-HD-NG
PthXo3
NI-HG-NI-HG-NI-NI-NI-HD-NN-HD-HD-HD-NG-HD-NG-NI-HD-HD-NN-NS-NI-NN-NN-NG-NN-HD-N*-NS-N*

PthA1
NI-N*-NI-N*-NI-HD-HD-N*-NI-HD-NI-HD-N*-NI-HD-HD-NG
PthA2
NI-HD-NI-HD-NI-HD-HD-NG-HD-NG-NG-NG-NG-NI-NI-NG
PthA3
NI-HD-NI-HD-NI-NG-HD-NG-NG-NG-NI-NI-NI-NI-HD-NG
PthA4
NI-N*-NI-NI-NI-HD-HD-NG-HD-NG-NG-NG-NG-NS-HD-HD-NG-NG

Hax2
NN-IG-NG-NI-NG-NG-HD-NG-HD-NI-HD-NI-HD-NG-HD-NG-HD-HD-NG-NG-NI-NG

Known TAL effectors of *Xanthomonas* strains

Xoo PXO99^A:
AvrXa27, PthXo1, PthXo6, PthXo7, Tal2a, Tal4, Tal5a, Tal6a, Tal7a, Tal7b, Tal8a, Tal8b, Tal9a, Tal9b, Tal9d, Tal9e

Xoc BSL256:
Avrpth3, Avrpth14, XOCORF_0460, XOCORF_0463, XOCORF_0473, XOCORF_1565, XOCORF_1566, XOCORF_1567, XOCORF_1570, XOCORF_1572, XOCORF_1583, XOCORF_1704, XOCORF_1709, XOCORF_1716, XOCORF_2007, XOCORF_2009, XOCORF_2010, XOCORF_2480, XOCORF_2485, XOCORF_2857, XOCORF_2892, XOCORF_4025, XOCORF_4248, XOCORF_4250, XOCORF_4384, XOCORF_4392, XOCORF_4399, XOCORF_4531

Xoo BAI3:
TalC

Xoo MAF311018:
XOO1132_MAFF, XOO1134_MAFF, XOO1136_MAFF, XOO1138_MAFF, XOO1996_MAFF, XOO1998_MAFF, XOO2001_MAFF, XOO2127_MAFF, XOO2129_MAFF, XOO2158_MAFF, XOO2160_MAFF, XOO2864_MAFF, XOO2865_MAFF, XOO2866_MAFF, XOO2868_MAFF, XOO4014_MAFF

Xac 306:
PthA1, PthA2, PthA3, PthA4

GUS assays and reporter constructs

Constructs were done similar as described previously [5]. The minimal *pBs4* promoter was PCR amplified and inserted into pENTR/D-TOPO (Invitrogen) with AvrXa10 target boxes at the 5' end. Promoters were recombined into the binary vector pGWB3 [6] containing a promoterless β-glucuronidase (*uidA*) gene. AvrXa10 was expressed from pGWB2 [6]. ArtBs4 was constructed with RVDs (NG-NN-NG-NI-NG-NI-NG-NI-NI-HD-NG-NG-NG) to match a sequence (TTGTATATAACTTT) present in the minimal *pBs4* promoter using a modified Golden TAL Technology [7]. TAL effector genes were under control of the constitutive *35S* promoter.

β-glucuronidase (GUS) assays were done as described previously [5]. Briefly, to assay GUS reporter activity, *Agrobacterium tumefaciens* strains delivering TAL effector constructs and GUS reporter constructs were mixed 1:1, and inoculated into 5-7 week old *Nicotiana benthamiana* leaves with an OD600 of 0.8. For qualitative GUS assays, leaf discs were sampled two days post infiltration (dpi), incubated in X-Gluc (5-bromo-4-chloro-3-indolyl-β-D-glucuronide) staining solution [8], destained in ethanol, and dried with acetate foil. For quantitative assays, two leaf discs (0.9 cm diameter) were sampled 2 dpi and GUS activity was determined using 4-methyl-umbelliferyl-β-D-glucuronide (MUG) as substrate. Proteins were quantified using Bradford assays (BioRad). Data correspond to triplicate samples from different plants. Experiments were performed twice with similar results.

References

1. Meila-Predoviciu M (1999) Learning with Mixtures of Trees. Ph.D. thesis, Massachusetts Institute of Technology. URL `http://people.csail.mit.edu/mmp/thesis.pdf`.

2. Keilwagen J, Grau J, Posch S, Grosse I (2010) Apples and oranges: avoiding different priors in Bayesian DNA sequence analysis. BMC Bioinformatics 11: 149.

3. Buntine WL (1991) Theory refinement of Bayesian networks. In: Uncertainty in Artificial Intelligence. Morgan Kaufmann, pp. 52-62.

4. Heckerman D, Geiger D, Chickering DM (1995) Learning Bayesian networks: The combination of knowledge and statistical data. In: Machine Learning. pp. 197–243.

5. Boch J, Scholze H, Schornack S, Landgraf A, Hahn S, et al. (2009) Breaking the Code of DNA Binding Specificity of TAL-Type III Effectors. Science 326: 1509-1512.

6. Nakagawa T, Kurose T, Hino T, Tanaka K, Kawamukai M, et al. (2007) Development of Series of Gateway Binary Vectors, pGWBs, for Realizing Efficient Construction of Fusion Genes for Plant Transformation. Journal of Bioscience and Bioengineering 104: 34-41.

7. Geißler R, Scholze H, Hahn S, Streubel J, Bonas U, et al. (2011) Transcriptional Activators of Human Genes with Programmable DNA-Specificity. PLoS ONE 6: e19509.

8. Schornack S, Peter K, Bonas U, Lahaye T (2005) Expression Levels of avrBs3-Like Genes Affect Recognition Specificity in Tomato Bs4- But Not in Pepper Bs3-Mediated Perception. Molecular Plant-Microbe Interactions 18: 1215–1225.
